# Identification of Direct Target Genes Using Joint Sequence and Expression Likelihood with Application to DAF-16

**DOI:** 10.1371/journal.pone.0001821

**Published:** 2008-03-19

**Authors:** Ron X. Yu, Jie Liu, Nick True, Wei Wang

**Affiliations:** 1 Department of Chemistry and Biochemistry, University of California San Diego, La Jolla, California, United States of America; 2 Center for Theoretical Biological Physics, University of California San Diego, La Jolla, California, United States of America; 3 Department of Computer Science, University of California San Diego, La Jolla, California, United States of America; University of Glasgow, United Kingdom

## Abstract

A major challenge in the post-genome era is to reconstruct regulatory networks from the biological knowledge accumulated up to date. The development of tools for identifying direct target genes of transcription factors (TFs) is critical to this endeavor. Given a set of microarray experiments, a probabilistic model called TRANSMODIS has been developed which can infer the direct targets of a TF by integrating sequence motif, gene expression and ChIP-chip data. The performance of TRANSMODIS was first validated on a set of transcription factor perturbation experiments (TFPEs) involving Pho4p, a well studied TF in *Saccharomyces cerevisiae*. TRANSMODIS removed elements of arbitrariness in manual target gene selection process and produced results that concur with one's intuition. TRANSMODIS was further validated on a genome-wide scale by comparing it with two other methods in *Saccharomyces cerevisiae*. The usefulness of TRANSMODIS was then demonstrated by applying it to the identification of direct targets of DAF-16, a critical TF regulating ageing in *Caenorhabditis elegans*. We found that 189 genes were tightly regulated by DAF-16. In addition, DAF-16 has differential preference for motifs when acting as an activator or repressor, which awaits experimental verification. TRANSMODIS is computationally efficient and robust, making it a useful probabilistic framework for finding immediate targets.

## Introduction

One of the major goals in the post-genome era is to establish a connectivity diagram of transcription network, which requires identification of direct targets of transcription factors (TFs). One commonly used approach to detect regulatory interactions between TFs and genes is chromatin immunoprecipitation followed by microarray hybridization (ChIP-chip)[1], [2], which is a binding assay. However binding of a TF to regulatory sequences does not necessarily imply regulation of gene expression. Furthermore, the applicability of ChIP-chip analysis is limited by the availability of antibody against a TF of interest. Therefore, ChIP-chip experiment is often complemented by functional assays using gene microarray.

To determine genes that are regulated by a specific TF, the TF is constitutively activated or inhibited such that the target genes of the TF should have significant expression changes in most of these experiments, which we call transcription factor perturbation experiments (TFPEs)[3]. In TFPEs, a combination of thresholds, e.g. the least amount of fold change considered to be significant and the minimum number of experiments in which the gene expression changes are required to be significant, need to be pre-specified. However the choice of threshold values tends to be arbitrary. Thresholds are usually hand-picked on a case-by-case basis, depending on the data set. More importantly, direct and indirect targets of the TF cannot be discriminated by expression alone.

In this paper, we present a probabilistic model called TRANSMODIS (TRANScription MOdule DIScovery) which integrates sequence and expression information in target identification. The parametric model can remove the arbitrariness commonly associated with the selection of thresholds for gene expression change. Consideration about the presence or absence of a binding motif in promoters can help distinguish direct from indirect targets. Many motif finding algorithms, for example references [4]–[15], have been developed and the performance of motif finding algorithms has been steadily improving. We thus assume that the core binding motif of a TF of interest has been determined *a priori* and is provided as an input to TRANSMODIS. TRANSMODIS is not a motif finding algorithm rather it focuses on determining direct targets of a TF.

Several computational methods had been developed previously to identify direct targets of TFs. MARSMotif[16], [17] fits splines to gene expressions and determines motifs and genes regulated by the motif simultaneously. Beyer *et al.* [18] applied a Bayesian method to integrate various types of information to generate a list of putative targets of TFs in yeast. Their approach was not designed to identify targets of a TF in multiple microarray experiments. ARACNe[19] is an approach for reconstructing regulatory networks from a large number of expression profiles. It first identifies statistically significant gene-gene coregulations, and then eliminates indirect relationships, which are thought to be the weakest interactions within three-gene loops. The idea is that the remaining edges in the network should have a high probability of representing either direct regulatory interactions or interactions realized by post-transcriptional modifications. ARACNe is a novel approach; however it does not make use of any sequence data and its inferred gene-gene interactions are non-directional. Segal *et al.* [20], [21] built probabilistic models to search for genes showing similar expression patterns and also sharing common motif profiles. Their models were complex and the parameters of their models were learned iteratively via greedy search. Compared with the general scenario that Segal *et al.* were dealing with, TRANSMODIS handles a much simpler situation. As the core motif is given and the target genes of the TF of interest should show significant expression changes in most of the experiments, the search for optimal parameter values in TRANSMODIS is less likely to be trapped in local optima.

The intuition behind TRANSMODIS is that genes containing the consensus core motif of the TF as well as exhibiting consistent expression changes in all TFPEs are likely to be true direct targets. In TRANSMODIS, gene expressions are modeled by a two-component Gaussian mixture model and the binding site sequences are assumed to be generated from a multinomial distribution which is represented by a position specific weight matrix (PSWM). By maximizing the joint likelihood of sequence and expression, TRANSMODIS identifies a set of genes that have consistent and highly elevated expressions and high scoring putative binding sites.

TRANSMODIS is a generalization of MODEM[22], a model we developed previously that is applicable only to a single gene expression microarray or ChIP-chip experiment. Compared with MODEM, TRANSMODIS is less sensitive to noise in individual experiments because of the consistency requirement on gene expression level across multiple experiments. TRANSMODIS also adds an additional step to score genes that do not contain a copy of the consensus binding motif in their promoter regions.

Because consensus binding motif is not known for every TF and sets of TFPEs are limited, a true genome-wide verification of TRANSMODIS is not yet practical. Thus we validated the performance of TRANSMODIS on Pho4p, a TF in budding yeast *Saccharomyces cerevisiae*. A comparison with previously reported target genes and the target genes selected by the original authors who did the perturbation experiments showed that TRANSMODIS is a promising method for direct target identification and is expected to yield a low false discovery rate (FDR) in general. On a larger scale, TRANSMODIS was applied to a set of ChIP-chip data[8] and evaluated against two other methods. Since no complete list of targets of any TF is known, the comparison was based on positive prediction value (PPV), which is the portion of true positives in all findings. TRANSMODIS demonstrated better performance than the two other methods on a majority of the 81 TFs tested. We then applied TRANSMODIS to identify immediate targets of DAF-16, which is a critical TF influencing the lifespan of nematode *Caenorhabditis elegans*.

## Results

### 1. Validation of TRANSMODIS by simulation

We first validated TRANSMODIS on simulated data where the true targets were known. Each simulated data set consisted of 1000 genes and ten experiments. Out of the 1000 genes, ten were targets and the other 990 genes were non-targets. The expression values of non-target genes were identically and independently sampled from the standard normal distribution *N*(0,1). And those of targets were simulated from the normal distribution with a mean of three and a variance of one *N*(3,1). To make the problem more challenging, within each experiment, ten non-target genes were randomly selected to have their expressions drawn from the *N*(3,1) distribution of target genes and five target genes were randomly selected to have their expressions reduced by half.

The consensus binding motif was chosen to be *TGTTTAC*. All target genes had this core binding motif present in their upstream sequences except for two of the ten target genes, which had binding motifs that differed from the consensus binding motif in two nucleotides, namely, *TTTTAAC* and *AGTTTCC*. The upstream sequences of all non-targets were simply generated from the uniform background. Each upstream sequence was 600-nucleotide long.

A total of ten simulated data sets were generated and analyzed. The results are listed in Table 1. TRANMODIS showed a clear advantage over MODEM on the simulated data sets. With most data sets (9 out of 10), TRANSMODIS identified the complete set of true targets except for the fifth simulated data set, where TRANSMODIS missed one true target. TRANSMODIS had no false positives in all cases. MODEM, on the other hand, failed to find any target genes by the majority voting rule. Note that when MODEM was applied to an individual array, it did identify a list of targets; however since most of the genes on the lists were false positives, no gene (including true targets) made half of the lists. The number of true targets on most lists was between zero and two. Thus the simulation study showed that the gain of using information from all arrays all at once by TRANSMODIS was substantial.

**Table 1:** TRANSMODIS and MODEM results on ten simulated data sets.

Simulated data set	#1	#2	#3	#4	#5	#6	#7	#8	#9	#10
TRANMODIS	10/10*	10/10	10/10	10/10	9/9	10/10	10/10	10/10	10/10	10/10
MODEM on array 1	1/33	0/57	2/59	1/40	2/26	0/24	0/55	2/67	4/72	0/55
MODEM on array 2	0/31	1/22	1/27	2/32	0/48	2/41	0/38	2/46	2/37	0/19
MODEM on array 3	0/37	2/45	1/35	0/36	0/19	2/38	3/41	0/28	0/23	0/58
MODEM on array 4	2/50	0/38	2/26	0/38	1/34	0/54	2/47	0/23	2/41	0/50
MODEM on array 5	2/43	0/17	0/38	1/32	3/30	3/50	1/73	1/63	0/29	1/80
MODEM on array 6	1/28	1/29	1/40	2/30	1/71	1/29	0/38	1/26	3/46	2/42
MODEM on array 7	1/33	0/40	0/38	0/53	3/36	0/45	2/35	6/41	0/33	2/34
MODEM on array 8	3/32	0/58	1/39	0/29	2/32	0/36	0/56	0/30	2/50	0/31
MODEM on array 9	0/22	1/45	1/94	1/25	0/52	0/45	3/69	0/30	0/19	0/26
MODEM on array 10	1/26	1/43	0/43	2/32	0/58	0/32	1/33	1/32	1/61	0/57
MODEM (majority voting)	0/0	0/0	0/0	0/0	0/0	0/0	0/0	0/0	0/0	0/0

* The ratio A/B indicates that the method predicted a total of B genes as direct targets and out of these B genes, A genes were true targets.

### 2. Validation of TRANSMODIS in *Saccharomyces cerevisiae*

To further validate the model, TRANSMODIS was applied to identify immediate targets of Pho4p, a TF in model organism *Saccharomyces cerevisiae*. Multiple perturbation microarray experiments were done for Pho4p. The PHO regulatory system is one of the most well studied pathways in *Saccharomyces cerevisiae*. In a low phosphate (P_i_) concentration medium, the cyclin-dependent kinase (CDK) inhibitor Pho81p inactivates the Pho80p-Pho85p complex, leading to an accumulation of hypophosphorylated form of Pho4p in the nucleus and subsequent activation of phosphate responsive genes. In order to identify all genes involved in the phosphate response, Ogawa *et al.* [23] carried out eight microarray experiments, namely, low P_i_ vs. high P_i_ in WT (NBW7) exp 1, low P_i_ vs. high P_i_ in WT (NBW7) exp 2, low P_i_ vs. high P_i_ in WT (DBY7286), PHO4^c^ vs. WT, pho80Δ vs. WT, pho85Δ vs. WT, PHO81^c^ vs. WT exp 1 and PHO81^c^ vs. WT exp 2. Pho4p was active in each of these experiments and up-regulated expressions of its target genes. Ogawa *et al.* considered a set of 20 genes that showed at least a two-fold increase of expression in at least five out of the eight experiments as Pho4p targets. In contrast to the somewhat arbitrary criterion used by Ogawa *et al.*, TRANSMODIS provides a parametric model to remove this arbitrariness.

Using the known binding motif *CACGTGG* of Pho4p and the eight microarray experiments of Ogawa *et al.* as inputs, TRANSMODIS found 19 genes from the entire *Saccharomyces cerevisiae* genome (about 6000 genes) as Pho4p targets (Table 2 and Table S1). The 19-gene TRANSMODIS target list was nearly identical to the 20 genes identified by Ogawa *et al.* except for *YER038C*, which was dropped by TRANSMODIS. The *YER038C* gene is unlikely to be PHO-regulated because it does not contain the consensus Pho4-binding motif or variants in its promoter.

**Table 2:** Target genes selected using different approaches.

Gene	ORF	Ogawa *et al.*	TRANSMODIS	MODEM (average expression profile)	MODEM (individual arrays; majority rule)	MODEM (PHO4^c^ vs. WT)
**PHO11** *	**YAR071W**	√	√	√	√	√
**PHO5** *	**YBR093C**	√	√	√	√	√
**PHO89** *	**YBR296C**	√	√	√	√	√
PHM6	YDR281C	√	√	√	√	√
PPN1	YDR452W	√	√		√	√
**PHO8** *	**YDR481C**	√	√	√	√	√
PHM8	YER037W	√	√		√	
HIS1	YER055C	√	√			
HOR2	YER062C	√	√	√	√	
VTC1	YER072W	√	√		√	√
VTC2	YFL004W	√	√	√		√
**SPL2** *	**YHR136C**	√	√	√	√	√
**PHO12** *	**YHR215W**	√	√	√	√	√
VTC4	YJL012C	√	√	√	√	√
**PHO86** *	**YJL117W**	√	√		√	√
**PHO84** *	**YML123C**	√	√	√	√	√
PHM7	YOL084W	√	√			
CTF19	YPL018W	√	√	√	√	√
VTC3	YPL019C	√	√	√	√	√
KRE29	YER038C	√				
SWC3	YAL011W					√
YAR069C	YAR069C					√
YAR070C	YAR070C			√	√	√
KRE2	YDR483W			√		√
MNN1	YER001W			√		
ARO9	YHR137W			√	√	√
REC107	YJR021C					√
YJR039W	YJR039W					√
NUP85	YJR042W					√
PTK2	YJR059W			√		
CDA1	YLR307W					√
YLR402W	YLR402W					√
YML089C	YML089C					√
YMR291W	YMR291W			√		
YPL110C	YPL110C			√		
CTF4	YPR135W					√
**PHO81** *	**YGR233C**					

* The nine genes that were previously reported to be under PHO regulation prior to the study of Ogawa *et al.* [23]

**Figure 1:**
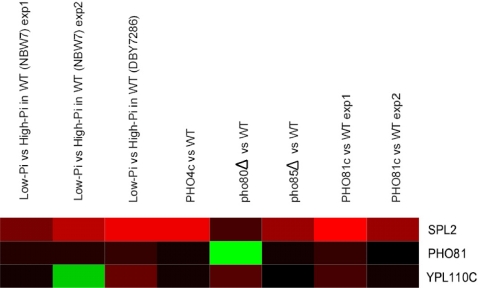
Comparison between the expression profiles of *PHO81* and its two homologs *SPL2* and *YPL110*C in the eight TFPE experiments of Pho4p. Red and green colors represent up- and down-regulation, respectively. The brightness of the color is proportional to the absolute expression ratio.

There were nine genes reported to be PHO-regulated prior to the study of Ogawa *et al.* These nine genes were *PHO11*, *PHO5*, *PHO89*, *PHO8*, *SPL2*, *PHO12*, *PHO86*, *PHO84* and *PHO81* [23]–[28]. All of them except *PHO81* were correctly identified as targets by both Ogawa *et al.* and TRANSMODIS. A heatmap of the expression profiles of *PHO81* and its two homologs *YPL110C* and *SPL2* is shown in Figure 1. The heatmap reveals that *SPL2* had a consistently higher differential expression in all experiments (an average increase of 16-fold) than *PHO81* and *YPL110C* (an average increase of 1.6-fold and 2-fold respectively) (p-value = 0.015 from two-sample t-test) (Figure 1). Indeed, both Ogawa *et al.* and TRANSMODIS identified *SPL2* as a Pho4p target. Based on the gene expression data, the selection of *SPL2* and the omission of *PHO81* and *YPL110C* by TRANSMODIS are consistent with one's intuition.

TRANSMODIS is an extension to MODEM, which was developed for analyzing a single microarray experiment. To compare the performance of TRANSMODIS with that of MODEM, we applied MODEM in two different ways on this data set. The first approach was to calculate the average expression of each gene in all experiments and apply MODEM to this “single” array of averaged expressions. The second approach was to apply MODEM on all eight expression data separately and then select target genes using majority voting (Table 2). We have also listed the MODEM result on a single *PHO4* mutation experiment PHO4^c^ vs. WT, in which the Pho4p was constitutively active in Table 2.

One of the known targets, *PHO81*, was missed by all approaches because of the weak evidence in the expression data (Figure 1). The eight other earlier known targets were successfully identified by all approaches. Only *PHO86* was missed when MODEM was run on the averaged expression profile of all arrays. It is not surprising that TRANSMODIS was more stringent than MODEM, identifying fewer targets than MODEM. The average number of target genes found by MODEM from an individual experiment of Ogawa *et al.* was 32. By requiring consistent up-regulation in all experiments, TRANSMODIS can filter out non-targets that would otherwise be erroneously identified from a single array analysis. At the same time, being less sensitive to random noise in individual experiments, TRANSMODIS can recover some of the true targets that would otherwise be missed by MODEM.

Different from MODEM, TRANSMODIS has an additional step of scoring promoter sequences that do not contain the consensus core motif (up to a certain number of allowed mismatches). Upon evaluation of such a gene without the core motif, if the probability of being a true target using the learned model parameters is greater than 0.5, TRANSMODIS will tag this gene as a target as well. For example, TRANSMODIS identified *PHM7* as a Pho4p target; the putative binding site in *PHM7* was found to be *CAAGTGC*, which differs from the consensus binding motif in two nucleotides and therefore was not evaluated by MODEM.

### 3. Comparative assessment of TRANSMODIS

There is only a limited number of multiple perturbation experiments publicly available for the same TFs. In order to assess the performance of TRANSMODIS on a genomic data set, we applied it to the ChIP-chip data of 204 TFs[8]. The ChIP-chip experiments were done under different conditions for a portion of the 204 TFs. There are 26 and 15 TFs for which ChIP-chip experiments were done under 3 and >3 conditions respectively. Since the TFs were not necessarily active under each of these conditions and the number of experiments was small, we could not blindly apply TRANSMODIS to experiments available for a TF. We therefore analyzed each ChIP-chip experiment separately and manually selected the experiment that satisfied the following two criteria: there is a significant motif identified by REDUCE[11] in the experiment and the enriched functions of the identified target genes are consistent with those of the TFs.

We compared the performance of TRANSMODIS with two other methods for identifying TF binding. The first one is a Bayesian method that integrates diverse information to predict TF binding in yeast[18] and the second one is an error model developed by Young and colleagues[2]. Since no complete list of targets for any TF is available, sensitivity and specificity cannot be calculated for any of these methods. Therefore, we computed PPV, the portion of true positives in the total predictions. The true positives were taken from three databases: TRANSFAC, SCPD and YPD. We compared the results of the three methods on 81 TFs that had at least one target gene known in the literature and on which the Bayesian method made predictions (Table 3).

**Table 3:** Comparison between TRANSMODIS and two other methods for target gene identification on the set of ChIP-chip data by Harbison *et al.* [8].

TF	Known targets	Total number of predictions			Number of predictions known to be true			PPV		
		TRANSMODIS	Bayesian	Error model	TRANSMODIS	Bayesian	Error model	TRANSMODIS	Bayesian	Error model
ABF1	30	240	176	267	9	5	5	0.038	0.028	0.019
ACE2	8	85	335	92	2	2	2	0.024	0.006	0.022
ADR1	10	189	20	35	1	0	0	0.005	0	0
ARG80	8	16	7	16	3	2	3	0.188	0.286	0.188
ARG81	8	17	20	28	3	4	4	0.176	0.200	0.143
ARO80	2	12	32	27	2	2	2	0.167	0.063	0.074
ASH1	1	21	10	0	0	0	0	0	0	NA
BAS1	13	41	147	41	8	10	8	0.195	0.068	0.195
CBF1	11	86	252	281	3	7	5	0.035	0.028	0.018
CIN5	1	117	169	153	0	0	0	0	0	0
CUP9	2	35	6	21	1	1	1	0.029	0.167	0.048
DAL80	22	49	8	13	0	0	0	0	0	0
DAL81	10	114	79	96	7	5	7	0.061	0.063	0.073
DAL82	8	54	93	59	6	8	6	0.111	0.086	0.102
FKH1	1	167	116	142	0	0	0	0	0	0
FKH2	2	121	353	122	2	2	2	0.017	0.006	0.016
FZF1	1	35	5	17	0	0	0	0	0	0
GAT1	4	124	41	27	3	1	1	0.024	0.024	0.037
GCN4	57	68	169	75	23	32	22	0.338	0.189	0.293
GCR1	20	42	55	15	0	5	2	0	0.091	0.133
GCR2	9	47	43	56	4	5	4	0.085	0.116	0.071
GLN3	31	118	141	68	16	16	11	0.136	0.113	0.162
HAC1	5	10	56	15	1	3	1	0.100	0.054	0.067
HAL9	1	33	15	28	0	0	0	0	0	0
HAP1	14	149	189	151	10	9	10	0.067	0.048	0.066
HAP2	30	23	54	21	2	2	2	0.087	0.037	0.095
HAP3	27	10	19	30	1	2	2	0.100	0.105	0.067
HAP4	27	74	170	77	7	9	7	0.095	0.053	0.091
HAP5	25	13	24	12	1	0	0	0.077	0	0
HSF1	16	71	122	102	12	12	13	0.169	0.098	0.127
IME1	15	20	1	0	0	0	0	0	0	NA
INO2	20	33	62	48	5	10	7	0.152	0.161	0.146
INO4	18	31	64	37	9	13	9	0.290	0.203	0.243
IXR1	1	9	2	28	0	0	0	0	0	0
LEU3	7	19	61	24	6	6	4	0.316	0.098	0.167
MAC1	8	8	47	18	3	4	4	0.375	0.085	0.222
MBP1	38	121	394	61	15	25	8	0.124	0.063	0.131
MCM1	32	92	240	107	18	20	16	0.196	0.083	0.150
MET28	1	20	1	17	0	0	0	0	0	0
MET4	9	25	76	28	4	5	1	0.160	0.066	0.036
MIG1	29	10	67	22	1	8	2	0.100	0.119	0.091
MOT3	4	22	11	8	0	0	0	0	0	0
MSN1	1	114	1	5	0	0	0	0	0	0
MSN2	36	154	199	47	11	17	4	0.071	0.085	0.085
MSN4	33	115	163	71	8	13	4	0.070	0.080	0.056
PDR1	15	323	108	8	4	4	0	0.012	0.037	0.000
PDR3	9	8	39	21	1	2	1	0.125	0.051	0.048
PHO2	19	33	2	33	1	0	1	0.030	0	0.030
PHO4	24	72	82	31	4	8	7	0.056	0.098	0.226
PPR1	4	15	24	28	0	2	0	0	0.083	0
PUT3	2	14	66	90	1	2	0	0.071	0.030	0
RAP1	35	291	196	0	17	13	0	0.058	0.066	N/A
RCS1	11	39	183	261	7	10	0	0.179	0.055	0
REB1	21	278	313	0	4	4	0	0.014	0.013	N/A
RFX1	5	12	57	25	2	4	2	0.167	0.070	0.080
RGT1	6	9	1	0	1	1	0	0.111	1.000	N/A
RIM101	4	115	27	7	0	0	0	0	0	0
RME1	2	29	66	40	1	1	0	0.034	0.015	0
ROX1	13	104	94	6	1	2	0	0.010	0.021	0
RPH1	1	25	68	8	0	1	0	0	0.015	0
RPN4	7	144	212	101	4	7	4	0.028	0.033	0.040
RTG3	5	26	47	37	4	4	4	0.154	0.085	0.108
SIP4	2	9	69	21	1	2	1	0.111	0.029	0.048
SKN7	21	187	201	190	8	6	6	0.043	0.030	0.032
STE12	78	60	567	63	24	34	25	0.400	0.060	0.397
STP1	1	60	117	72	1	1	0	0.017	0.009	0
SUM1	2	81	110	60	1	0	1	0.012	0	0.017
SUT1	1	95	73	69	0	0	0	0	0	0
SWI4	14	105	271	161	5	6	4	0.048	0.022	0.025
SWI5	11	46	203	120	3	7	5	0.065	0.034	0.042
SWI6	44	118	430	158	10	19	10	0.085	0.044	0.063
TEC1	44	62	46	43	3	0	0	0.048	0	0
THI2	8	34	67	47	5	8	7	0.147	0.119	0.149
UGA3	3	9	42	32	2	2	0	0.222	0.048	0.000
UME6	40	286	239	134	18	18	10	0.063	0.075	0.075
XBP1	5	65	50	77	1	1	1	0.015	0.020	0.013
YAP1	39	25	314	72	5	11	7	0.200	0.035	0.097
YAP6	1	15	242	60	1	0	1	0.067	0	0.017
YHP1	1	42	9	20	0	0	0	0	0	0
YRR1	4	66	3	23	0	0	0	0	0	0
ZAP1	12	22	62	22	4	9	4	0.182	0.145	0.182
Average	14.4	72.8	111.3	58.6	4.3	5.6	3.5	0.086	0.066	0.063

The cutoff of the error model is set to 0.001, as suggested by the original authors[2].

On average, the Bayesian method had the most predictions while the error model had the least. The average PPV for the TRANSMODIS, the Bayesian method and the error model were 8.58%, 6.57% and 6.32%. More specifically, TRANSMODIS performed better than the Bayesian method and the error model on 44 and 46 TFs respectively, and TRANSMODIS performed worse than the other two methods on 22 and 13 TFs respectively. The PPVs are small for all three methods, which is probably due to the fact that only a small set of conditions was tested in the ChIP-chip experiments. It also highlights the need to continuously improve target identification methods.

### 4. Identification of genes involved in ageing

Encouraged by the success of TRANSMODIS on finding direct targets of TFs in *Saccharomyces cerevisiae*, we applied it to tackle a more challenging problem, namely the identification of direct targets of DAF-16 in nematode *Caenorhabditis elegans*. DAF-16 is a TF playing critical roles in worm ageing. The mechanism of ageing remains to be an important and unsolved mystery. Whereas the normal lifespan of an adult worm is only two to three weeks, individuals carrying mutations that decrease insulin/insulin-like growth factor 1 (IGF-1) signaling can live twice as long[29]. Mutations in gene *daf-2*, which is predicted to encode an insulin/IGF receptor ortholog, together with a downstream TF, *daf-16*, can increase lifespan significantly. DAF-2 negatively regulates the activity of DAF-16, a FOXO-family TF.

Identifying direct targets of DAF-16 can shed light on the functional mechanism of DAF-16 at influencing lifespan. Lee *et al.* [30] took a comparative genomics approach to identify orthologous genes containing the conserved DAF-16 binding sites in their promoter sequences and Oh *et al.* [31] used chromatin immunoprecipitation (ChIP) followed by cloning to search for direct downstream targets of DAF-16. Lee *et al.* found that the expression of 7 genes were controlled by DAF-16 while Oh *et al.* chose to study 33 genes out of 103 candidates and 18 genes showed significant (either up or down) expression changes in a *daf-16* dependent manner. The results of these studies were useful but the number of direct targets identified was limited. To identify genes that are regulated by the DAF-2 pathway and investigate their roles in the ageing process, Murphy *et al.* [32] deduced the *daf-2* and *daf-16* activity using RNAi and analyzed the resultant gene expression profiles using cDNA microarrays. First, genes with a minimum of fourfold expression change were selected by hierarchical clustering of 60 arrays (5 mutant arrays plus 55 time course arrays); in addition, genes showing highly consistent expressions, regardless of the amount of fold change, were also included. Then based upon the p-values obtained from SAM[33] and a visual inspection of genes for genes that were more overly expressed than the others, a top group of 58 genes was chosen to be further validated for their influence on lifespan[32].

The gene expression microarray experiments conducted by Murphy *et al.* [32] were functional assays and had multiple time points. We re-analyzed the data using TRANSMODIS to automatically identify the direct targets of DAF-16 without arbitrary thresholds and human involvement. We pooled together the time course data, which consisted of an early adult time course (ten time points from 0–48 h of adulthood) and a longer time course (ten time points from 0–192 h of adulthood), on worms exposed to *daf-2* RNAi and worms exposed to *daf-16* and *daf-2* RNAi. Arrays at 0h time point were left out of the analyses and we also discarded eight arrays with a high percentage of missing data. It left us with a set of twenty eight arrays. The numbers of *daf-2*(RNAi) treatments and *daf-2*(RNAi);*daf-16*(RNAi) treatments were approximately equal (15 versus 13). We retrieved 1kb upstream sequence of the translational start site of each ORF from WormBase[34].

Using the twenty eight time course gene expression arrays, the upstream sequence data, and the binding motif *TRTTTAC* defined by Murphy *et al.* [32], TRANSMODIS was run twice to the same data set with signs inverted in the second run, giving two classes of genes. Following the nomenclature defined in Murphy *et al.*, class 1 genes are genes that were induced in *daf-2*(RNAi) animals but repressed in *daf-2*(RNAi)*;daf-16*(RNAi) animals, and class 2 genes are the opposite genes which were repressed in *daf-2*(RNAi) animals but induced in *daf-2*(RNAi);*daf-16*(RNAi) animals. Class 1 and class 2 genes are candidate genes that extend and shorten worm lifespan respectively.

**Figure 2:**
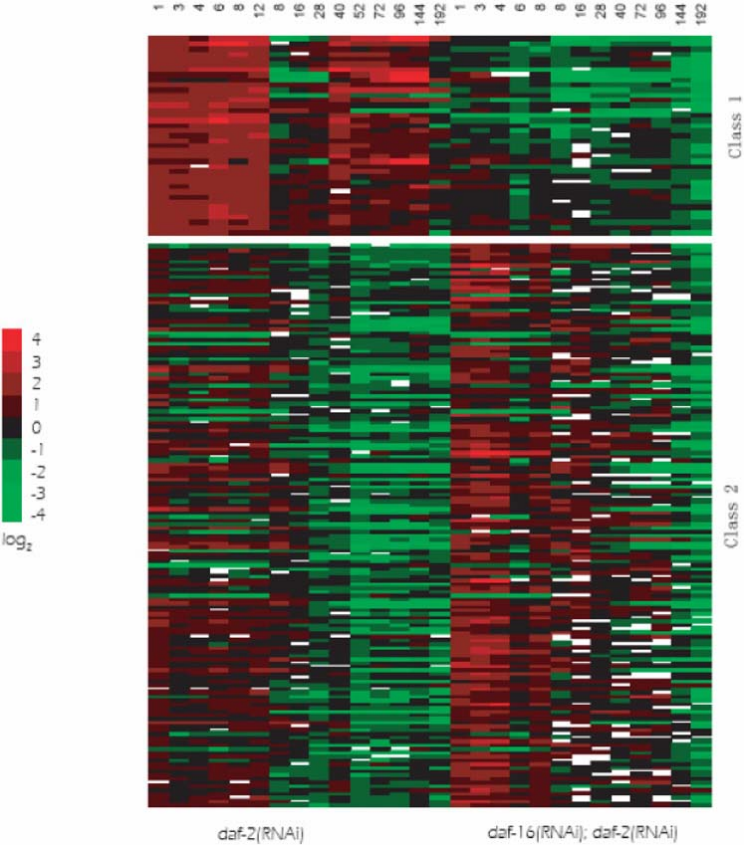
Expression profiles of class 1 and class 2 direct targets of DAF-16 in *Caenorhabditis elegans* identified by TRANSMODIS.

TRANSMODIS identified 39 class 1 genes and 150 class 2 genes (Figure 2, Table S2 and Table S3), compared with 263 class 1 genes and 251 class 2 genes that were found by Murphy *et al.* [32] using hierarchical clustering. Twenty of the TRANSMODIS predictions are in common with the 58 genes in Murphy *et al*. Furthermore the two lists of class 1 genes share 34 genes and the two class 2 gene lists overlap with 44 genes. The amount of overlap is statistically significant. Hierarchical clustering by itself cannot distinguish between direct and indirect targets. That was why Murphy *et al.* [32] used other criteria to prioritize their target list. TRANSMODIS provided a systematic and automatic target selection procedure that can be used in place of the original authors' method which needed human involvement.

There was no significant overlap between the targets found by TRANSMODIS and the two previous studies of Lee *et al.* [30] and Oh *et al.* [31]. The target genes identified by Lee *et al*. and Oh *et al.* did not have consistent significant expression changes in the time course experiments of Murphy *et al*. It could be that those genes are regulated by DAF-16 transiently or only at a specific temporal stage. For example, the expression of ZK593.4 was significantly upregulated in the short time course experiments of *daf-2* RNAi at 1, 3, 4, 6, 8 and 12 hour time points, but showed almost no change in the long time course experiments of *daf-2* RNAi. In the double *daf-2;daf-16* RNAi knock-down experiments, ZK593.4 had significant down-regulation only at the first three time points. Such a pattern was not unique to ZK593.4 and was observed for thousands of genes and hence it is hard, if not impossible, to pick out direct targets of DAF-16 exhibiting this particular pattern. The targets identified by TRANSMODIS could be complementary to the previous studies of Lee *et al.* [30] and Oh *et al.* [31].

**Figure 3:**
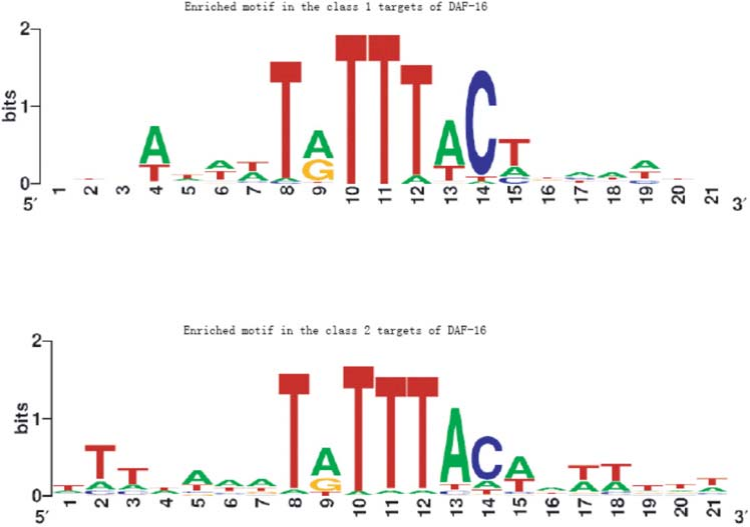
Enriched motifs in the class 1 and class 2 target genes of DAF-16. The *x* axis is the position and the *y* axis is the log_2_ ratio between the target and non-target weight matrices.

The extended motifs (the core motif plus immediate flanking regions) of the TRANSMODIS targets are shown in Figure 3 and the extended motifs of the two classes differ significantly at the flanking regions. The class 1 genes seem to prefer *GSGAGNNTRTTTACTBCANCG* (the core motif is underlined) while the class 2 genes seem to prefer *STCGACRTRTTTACAGNTSGS*. It was suggested that DAF-16 can function both as an activator and a repressor[30], [32]. The direction of regulation by DAF-16 may depend on cooperation between DAF-16 and other TFs binding to the same promoter[30], [32]. Our finding suggests the possibility that the binding sites of the other TFs may partially overlap with that of DAF-16. We therefore hypothesize that the extended motifs of the two target classes are recognized by TFs that function side by side with DAF-16 in a competitive or cooperative manner. This hypothesis can be tested experimentally by using immobilized DNA segments to pull down the co-factors.

We searched for enriched motifs in the 1 kb upstream sequences of TRANSMODIS targets using MobyDick[35], a dictionary motif finding algorithm. The MobyDick algorithm found approximately 300 motifs in each class of targets. We clustered these motifs based on their similarities and evaluated the significance of their occurrences using bootstrap. Among the class 1 targets, *AGTTCC*, *CTCCACC*, *CTGATAAG* and *CTTATCA* were significantly enriched (p-values<0.01, unadjusted for multiple testing). The p-value of a motif was computed as the probability of observing the same or larger number of occurrences of that motif in a random set of genes, which was a bootstrap sample without replacement from the entire *Caenorhabditis elegans* genome. We took 10,000 bootstrap samples to compute the p-values. The motif *CTTACTA* matched the binding motif of GATA family of TFs documented in WormBase[34] and was also identified as an enriched motif by Murphy *et al.* [32]. Murphy *et al.* pointed out in their paper that the motif *cttatca* might be bound by a TF that cooperates with DAF-16. Among the TRANSMODIS class 2 genes, the following motifs were significantly enriched: *AGATKAGR*, *CTGATAAG* and *CTTATCA*. We then scanned the 2000 bp upstream region of translational start site of TRANSMODIS class 1 and class 2 homolog genes (the best BLAST matches) in human. The motif *CTGATAAG* was found to be enriched in the class 1 human genes as well (bootstrap p-value = 0.0061), which suggested that this motif may have functional roles. The other motifs had failed to make the 0.01 p-value cutoff. It is not clear at this point whether *CTGATAAG* is an extended reverse variant of the canonical GATA motif *TGATAAG* or a binding site for another TF. There are 11 GATA factors encoded in the *Caenorhabditis elegans* genome. The deviation of *CTGATAAG* from the canonical GATA motif implies that, if it is indeed bound by a GATA factor, then only a subset of GATA factors specifically bind to this motif and cooperate with DAF-16 to regulate the class 1 genes. Since oxidoreductases are enriched in the class 1 genes (see below) and GATA factors MED-1 and MED-2 are known to be involved in oxidative stress response mediated by SKN-1[36], MED-1 and MED-2 should be the first TFs to be investigated.

To understand the mechanism of DAF-16 at affecting lifespan, we examined enriched molecular functions for the two classes of target genes. On the Murphy *et al.* class 1 and class 2 genes, the GO term analysis showed that the class 1 genes were enriched for oxidoreductase activities and the class 2 genes were enriched for peptidase activities. The target genes selected by TRANSMODIS had significant overlap with the Muphy *et al.* genes for both classes. However while there were still many oxidoreductases among the TRANSMODIS class 1 genes, the TRANSMODIS class 2 genes were no longer enriched for peptidase activities. Therefore there were slight changes in the GO term analysis results between the two sets of class 2 genes.

Among the twenty TRANSMODIS class 1 genes that had gene ontology annotations, nearly half of them (9 out of 20) were oxidoreductases (the Bonferroni corrected p-value was about 10^−4^). Numerous correlations between oxidative stress resistance and longevity have been described[37], consistent with the observation that *daf-2* RNAi worms lived significantly longer than wild types. This observation also highlights the regulatory role of DAF-16 on oxidoreductases to extend lifespan. The nine oxidoreductases are C30G12.2, R09B5.6, C06B3.4, W06D12.3, C06B3.5, B0213.15, K12G11.3, F11A5.12 and K07C6.4. Murphy *et al.* [32] had examined five of them, namely C06B3.4, B0213.15, K12G11.3, F11A5.12 and K07C6.4, on affecting animal lifespan using RNAi. Knocking down the activities of all but B0213.15 extended lifespan, though not significantly[32]. No significant biological processes or compartments were found, implying that the oxidoreductases are involved in many different processes. Combined with the functional study in [32], the GO term analysis suggested that the effects of oxidoreductases on ageing might be cooperative/collective and this is why mutations of their upstream regulators, e.g. DAF-2 and DAF-16, can significantly extend lifespan. TRANSMODIS identified 150 class 2 genes, involved in a diverse array of biological processes and functions. A significant portion of the genes (12 out of 63 annotated genes) are involved in macromolecule metabolism but the p-value was not significant at all. The most enriched biological processes were phosphate transport (13 out of 63 genes, p-value = 10^−10^) and ion transport (15 out of 63 genes, p-value = 10^−9^). The molecular functions of the class 2 genes with a p-value<0.01 were being structural constituents of cuticle (12 genes, p-value = 10^−10^) and structural molecules (14 genes, p-value = 10^−5^). These observations suggest possible functional roles of DAF-16 on affecting lifespan that have not yet been well studied.

## Discussion

TRANSMODIS is a probabilistic model for predicting direct targets from binding motif, sequence data, expression data and ChIP-chip experiments. The probabilistic framework removes arbitrary cutoffs in target selection procedures and allows integration of data coming from various sources. Compared with other criteria for identifying targets, TRANSMODIS is usually more stringent by requiring consistent and significant expression fold changes across all experiments.

The methodology was validated on a set of TFPEs perturbing the activity of Pho4p in *Saccharomyces cerevisiae*. TRANSMODIS had successfully recovered a majority of previously known direct targets, i.e. the nine genes that were reported to be PHO-regulated prior to the study of Ogawa *et al.* Because we do not know the total number of true targets of Pho4p, it is difficult at the current stage to give sensitivity and specificity analyses of TRANSMODIS. To assess the performance of TRANSMODIS, we applied TRANSMODIS and two other methods (a Bayesian method[18] and an error model[2]) on a set of 81 TFs in *Saccharomyces cerevisiae*. Using PPV as a measure of efficiency and accuracy, TRANSMODIS performed better than the Bayesian method and the error model on 44 and 46 TFs, and performed worse than the other two methods on 22 and 13 TFs, respectively.

Using simulated data sets, it was shown that TRANSMODIS could recover nearly every target gene every time and had few false positives; whereas MODEM, a previously developed method which is applicable to a single experiment, failed to find any target genes on the same data sets. Therefore, TRANSMODIS, though an extension of MODEM, was much more effective at identifying targets than MODEM when multiple arrays were available. If TRANSMODIS is fed a random motif, it can still make target predictions provided that the expression data is unaltered. This is due to the fact that true consensus binding motifs are usually short and degenerate, hence contributing less information than genomic expression data, especially when that data is combined from several experiments.

Some true targets can be missed by TRANSMODIS if the true targets had inconsistent induction in all experiments. The reason can be biological (e.g., transient regulation by the TF or combinatorial regulation of several TFs) or technical (e.g., systematic error or noise of microarray experiments). Nevertheless, the result of TRANSMODIS would be consistent with one's intuition given the data.

The usefulness of TRANSMODIS was demonstrated in the identification of immediate targets of DAF-16, a critical TF in *Caenorhabditis elegans* that regulates ageing. TFPE experiments are functional assays and are commonly used by researchers to identify targets of a TF, particularly in higher organisms. TRANSMODIS identified target genes that showed DAF-16 dependent expression changes, and expanded the list of known DAF-16 targets. An interesting finding of our analysis is that the flanking sequences of the core motif recognized by DAF-16 differ dramatically in the two classes of targets with opposite effects on lifespan. The observation may provide a clue to the TFs that cooperate with DAF-16 to specifically regulate the two classes of genes. We also found several putative binding motifs for the co-factors of DAF-16 in regulating lifespan. In particular, GATA factors may play important roles in regulating class 1 genes.

It is possible to obtain comparable results to TRANSMODIS by raising the cutoffs sometimes. However it is not clear how high the cutoffs should be set to in the absence of a guideline. If we require the induction ratio of target gene expression to be at least two-fold in at least six out of the eight Pho4p experiments done by Ogawa *et al.*, the target list will then shorten to fewer than 17 genes. So in order to yield a comparable target list, we probably would like to stick with the selection rule of requiring a marked up-regulation in five experiments for targets. Depending on the specific choice of the threshold, the final Pho4p target list is going to be of different length. For example, the target gene list consists of 20, 19 and 18 genes if the required cutoff is set to 2.1-fold, 2.2-fold, and 2.3-fold respectively. When the cutoff is raised from two-fold (the original threshold used by Ogawa *et al.*) to 2.1-fold, there is no change to the target list. When the cutoff is raised from 2.1-fold to 2.2-fold, gene *YER038C/KRE29* gets dropped and the target list becomes identical to the TRANSMODIS target list. Further increasing the cutoff to 2.3-fold drops gene *YOL084W/PHM7*, which is likely to be a true Pho4p target. Therefore even though it is possible to produce comparable results to TRANSMODIS by changing the thresholds, it is unclear how to find these thresholds and any choice would be arbitrary without an appropriate justification.

TRANSMODIS assumes that (1) the TF of interest has activities in all experiments; and hence the true immediate targets of a TF of interest ought to have consistent and significant expression changes in most if not all microarray experiments, and (2) the promoters of direct targets contain good matches to the consensus binding motif. These assumptions do not always hold. For example, the promoters of targets may contain motifs that could be bound by the TF but are not because of a lack of co-factors or an inaccessible chromatin structure. Or there can be a situation where only a subset of direct targets was upregulated because the TF recognizes different motifs under different conditions. In these situations, TRANSMODIS is not able to recover the full set of targets but only a subset of them.

In order to use TRANSMODIS, one has to supply a consensus binding motif, which is not always known in advance, especially in higher eukaryotic organisms. However as more biological knowledge is accumulated and deposited into databases such as TRANSFAC[38] and JASPAR[39], we believe that TRANSMODIS will find more applications in the future. A Java implementation of TRANSMODIS is available upon request. Or the users may choose to upload and analyze their microarray data at http://haedi.ucsd.edu/.

## Materials and Methods

### The parametric model of TRANSMODIS

The model contains two components: expression and sequence. Target genes should differ from non-targets in both expression levels and patterns of extended motifs. The expressions of targets and non-targets were modeled by a two-component Gaussian mixture distribution, and the nucleotide frequencies at each position of an extended binding motif were assumed to be multinomial which was represented by a position specific weight matrix (PSWM). The PSWM for non-target genes was the background nucleotide frequencies in the entire genome. Many methods for regulatory network reconstruction simply assume a uniform background distribution. However the uniform background assumption weakened the list of learned genes by TRANSMODIS by including an excess of false targets, especially on the *Saccharomyces cerevisiae* data. The model assumptions of TRANSMODIS are: (1) arrays are independent; (2) all arrays have the same mean and variance for targets and also the same mean and variance for non-targets, and (3) genes are independent from each other in terms of expression and upstream sequence composition. The maximum likelihood estimators (MLEs) of model parameters were computed via an expectation-maximization (EM) algorithm. Since the variances of target and non-target expression distributions are allowed to be unequal, unintuitive interpretation of expression data can occur (Figure S1). A procedure has been put in place to avoid making such incorrect inferences. A robust version of the formula for updating the variance of expression distribution of targets has also been investigated. The differences were found to be minimal when the true expression model was a two-component Gaussian mixture model (Figures S2 and S3, Table S4). Details as well as the derivation of the EM algorithm can be found in the supplementary materials.

Moderate deviations from the list of assumptions can be well tolerated by TRANSMODIS. Gross violations will result in a reduction of power in identifying true targets.

### The program

The inputs to TRANSMODIS are: (1) the 5′ upstream sequences of all genes in the genome; (2) multiple genome-wide microarray measurements, such as TF perturbation experiments (TFPEs)[3] or ChIP-chip experiments[1], [2] or a combination of both. The parametric framework allows ChIP-chip experiments to be incorporated into the model just as any other microarray experiments as long as the TF is activated under the ChIP-chip experimental conditions; and (3) the core DNA motif recognized by the TF, typically six to eight bases long. The core motif could have been known *a priori* or be identified by a motif finding algorithm.

The TRANSMODIS program consists of two steps. In the first step, the parametric model of TRANSMODIS is fitted to genes containing matches to the input core motif in their promoters to obtain MLEs via an EM algorithm (details can be found in the supplementary text (Text S1)). The matches do not have to be perfect matches; it is still considered a match if the nucleotide subsequence differs from the core motif in only one base pair. The reverse complement of the input core motif is also scanned for. If a promoter has multiple matched copies of the input core motif, all copies are extracted and aligned to create an initialization of the PSWM of the target genes. Then during iterations of the EM algorithm, the copy with the highest score according to the current estimate of the target PSWM is chosen as the putative transcription factor binding site.

In the second step of the TRANSMODIS analysis, genes that do not contain copies of the core motif (i.e. genes that were not used for the estimation of model parameters in the first step) have their promoters scanned for the core motif on both strands. If the probability of being a target is computed to be greater than that of being a non-target, the gene will be brought into the target list. No model parameters are estimated or modified during this step. The sole purpose of this second step is to catch potential true targets that lack a copy of the consensus binding motif and therefore would otherwise be overlooked if this step was not taken.

The output of TRANSMODIS are (1) two PSWMs, one for target genes and the other for non-targets. The weight matrices go beyond the core motif and cover the immediate flanking regions beside the core motif; and (2) the probability of being a true target for each gene. By default, genes are identified as targets if the probabilities are greater than 0.5.

TRANSMODIS is computationally efficient and converges fast. The running times on the Pho4p and Daf16p data sets were 2 minutes and 31 seconds and 8 minutes and 53 seconds respectively on a 2.4 GHz single processor computer with 512 KB of cache memory.

### GO term analysis

GO Term Finder[40] was used for the gene ontology analyses. The analyses were run on the annotation file submitted on March 21, 2006 for *Saccharomyces cerevisiae* and the annotation file submitted on March 20, 2006 for *Caenorhabditis elegans*. Bonferroni correction was used to adjust p-values for multiple testing.

## Supporting Information

Figure S1 Illustration of drawing invalid conclusions due to unequal variances. Two scenarios are depicted here: (A) target distribution has a greater mean and a greater variance and (B) target distribution has a greater mean and a smaller variance. In particular, in panel (A) the distributions are assumed to be N(0,1) and N(3,1.4) for non-targets and targets respectively. Then the target distribution curve lies above the non-target's for all expression values less than −7.5, thus making genes with small expression values (<−7.5) inappropriately identified as target genes instead of non-targets (e.g., for an expression value of −10, the ratio of conditional probabilities is as large as 700). In panel (B), the target and non-target distributions are assumed to be N(3,0.4) and N(0,1) respectively. Because of the smaller variance, the target distribution goes to zero faster than the non-target distribution does as expression level increases. For an expression value of 6, the odds of drawing such an expression value from the non-target over the target distribution is greater than 104. However it is incorrect to conclude that genes having expression values of 6 or greater are much more likely to be non-targets than targets.
(1.75 MB TIF)

Figure S2 Comparison of sensitivity between the two updating formulas for the standard deviation of target distribution.
(7.86 MB TIF)

Figure S3 Comparison of specificity between the two updating formulas for the standard deviation of target distribution. (Even though the one standard error bar is drawn above one, no actual specificity was ever greater than one.)
(7.92 MB TIF)

Table S1 (0.06 MB DOC)

Table S2 (0.10 MB DOC)

Table S3 (0.32 MB DOC)

Table S4 (0.02 MB DOC)

Text S1 Supplementary text
(0.24 MB DOC)
