# Supplementary figures and images for: Identification of Direct Target Genes Using Joint Sequence and Expression Likelihood with Application to DAF-16
Source: PLoS One. 2008 Mar 19;3(3):e1821. doi: 10.1371/journal.pone.0001821 (PMC2266795; doi:10.1371/journal.pone.0001821)

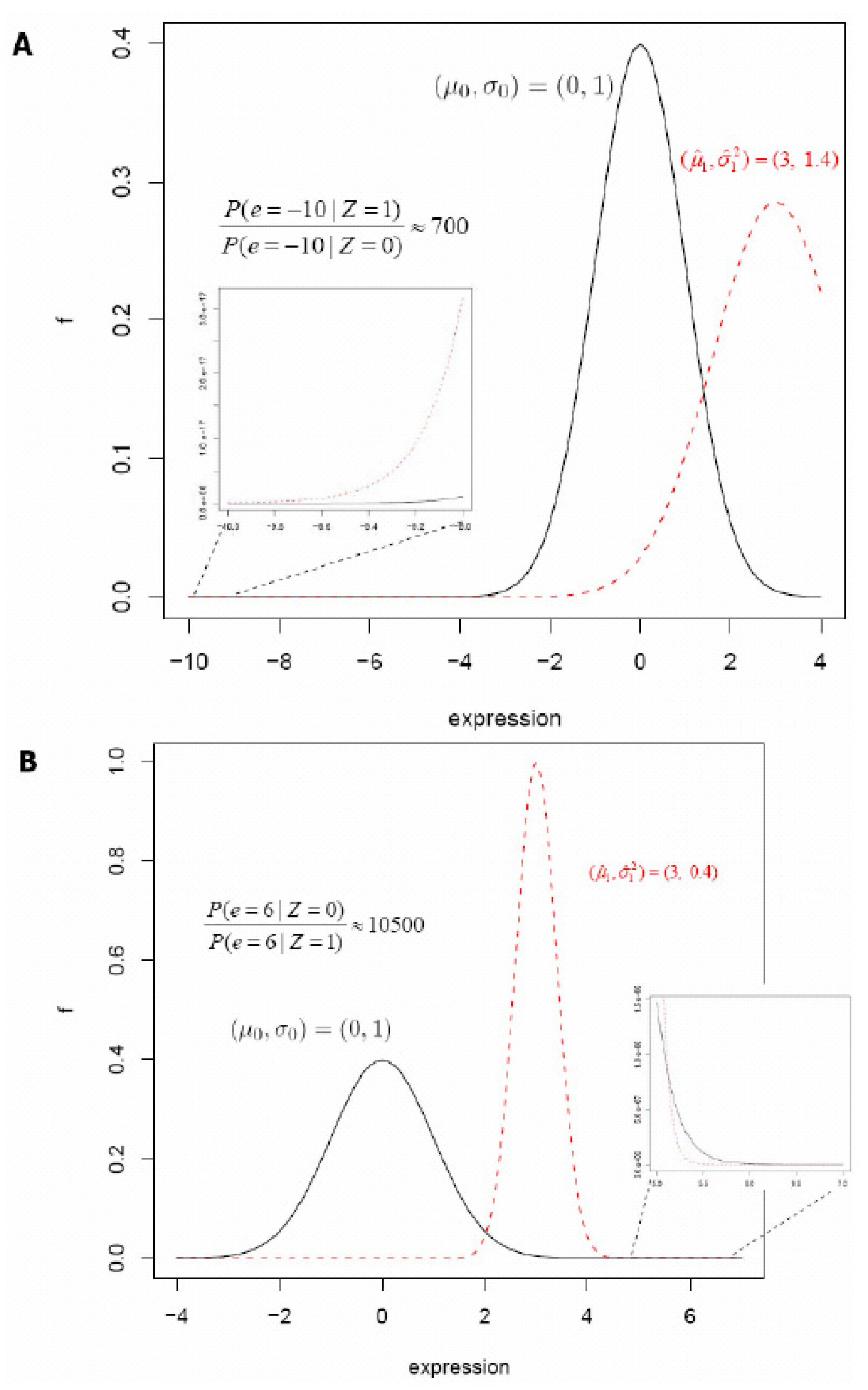

Supplement: Figure S1 — Illustration of drawing invalid conclusions due to unequal variances. Two scenarios are depicted here: (A) target distribution has a greater mean and a greater variance and (B) target distribution has a greater mean and a smaller variance. In particular, in panel (A) the distributions are assumed to be N(0,1) and N(3,1.4) for non-targets and targets respectively. Then the target distribution curve lies above the non-target's for all expression values less than −7.5, thus making genes with small expression values (<−7.5) inappropriately identified as target genes instead of non-targets (e.g., for an expression value of −10, the ratio of conditional probabilities is as large as 700). In panel (B), the target and non-target distributions are assumed to be N(3,0.4) and N(0,1) respectively. Because of the smaller variance, the target distribution goes to zero faster than the non-target distribution does as expression level increases. For an expression value of 6, the odds of drawing such an expression value from the non-target over the target distribution is greater than 104. However it is incorrect to conclude that genes having expression values of 6 or greater are much more likely to be non-targets than targets. (1.75 MB TIF) [file pone.0001821.s001.tif]

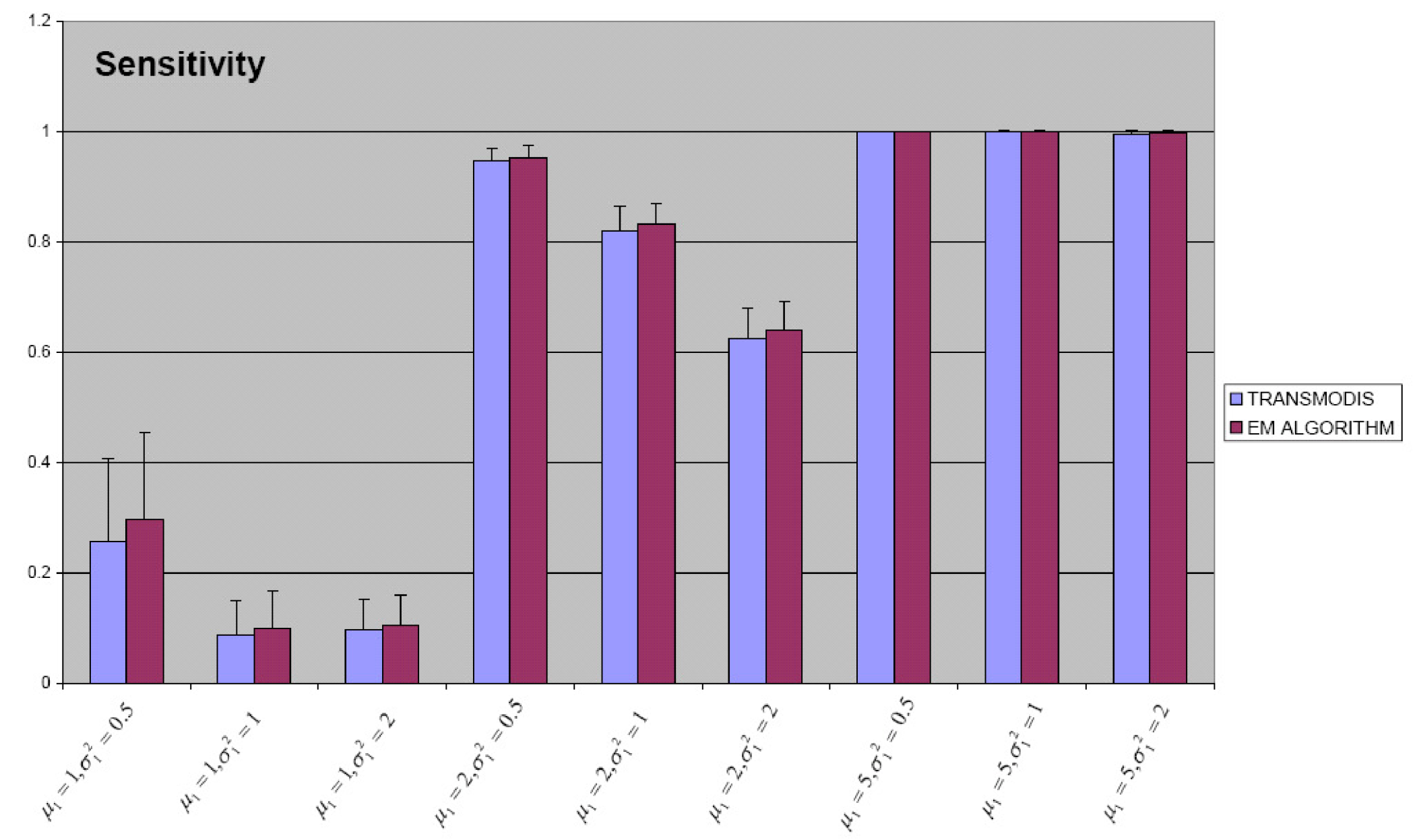

Supplement: Figure S2 — Comparison of sensitivity between the two updating formulas for the standard deviation of target distribution. (7.86 MB TIF) [file pone.0001821.s002.tif]

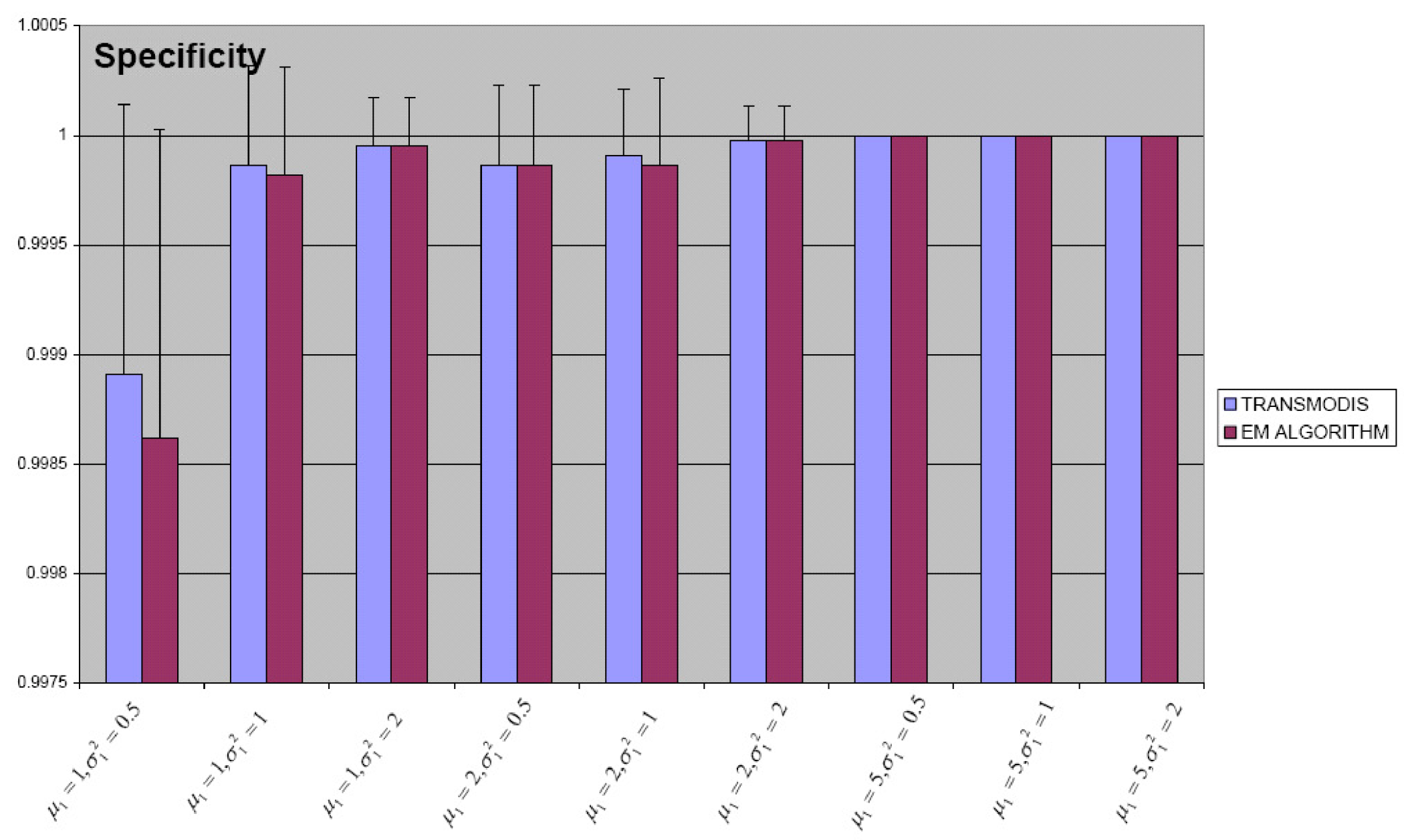

Supplement: Figure S3 — Comparison of specificity between the two updating formulas for the standard deviation of target distribution. (Even though the one standard error bar is drawn above one, no actual specificity was ever greater than one.) (7.92 MB TIF) [file pone.0001821.s003.tif]
