# Supplementary material for: Identification of Direct Target Genes Using Joint Sequence and Expression Likelihood with Application to DAF-16
Source: PLoS One. 2008 Mar 19;3(3):e1821. doi: 10.1371/journal.pone.0001821 (PMC2266795; doi:10.1371/journal.pone.0001821)
Supplement: Table S1 — (0.06 MB DOC) [file pone.0001821.s004.doc]

Table S1. Pho4p target genes identified by TRANSMODIS

| Gene ORF/Name | *p^*^* | Extended motif | A*^†^* | B*^†^* | C*^†^* | D*^†^* | E*^†^* | F*^†^* | G*^†^* | H*^†^* |
| --- | --- | --- | --- | --- | --- | --- | --- | --- | --- | --- |
| YAR071W/PHO11 | 1 | gcgttcacacgtgggtttaaa | 4.00 | 3.01 | 3.71 | 4.29 | 4.25 | 3.55 | 4.86 | 4.50 |
| YBR093C/PHO5 | 1 | gcactcacacgtgggactagc | 3.08 | 1.25 | 3.09 | 2.82 | 4.29 | 3.08 | 4.07 | 4.62 |
| YBR296C/PHO89 | 1 | aatgcagcacgtgggagacaa | 2.01 | 2.26 | 5.07 | 5.26 | 2.62 | 3.12 | 4.92 | 2.79 |
| YDR281C/PHM6 | 1 | tcgctgacacgtgggaggtgg | 1.37 | 0.87 | 3.40 | 3.00 | 3.04 | -0.29 | 3.62 | 0.97 |
| YDR452W/PPN1 | 1 | aaattaggacgtggtttatag | 2.60 | 1.25 | 0.78 | 1.76 | 1.95 | 1.14 | 2.29 | 1.98 |
| YDR481C/PHO8 | 1 | atcgctgcacgtggcccgacg | 1.71 | 0.87 | 1.80 | 1.55 | 1.90 | 1.32 | 2.17 | 2.01 |
| YER037W/PHM8 | 1 | tgtgaagcacgttgctgcccc | 0.54 | 0.16 | 1.97 | 0.33 | 1.53 | 1.31 | 2.20 | 1.97 |
| YER055C/HIS1 | 0.995 | ggtgactcacttggaagcttt | 1.35 | 0.57 | 0.75 | 1.28 | 1.66 | 0.79 | 1.55 | 1.32 |
| YER062C/HOR2 | 1 | tttacgtcacgtgggaggccc | 1.91 | 0.58 | 1.59 | 1.02 | 1.26 | 1.36 | 1.35 | 1.54 |
| YER072W/VTC1 | 1 | tccgagacacgtgctaatatc | 3.17 | 2.43 | 3.14 | 2.49 | 2.51 | 2.52 | 3.21 | 2.04 |
| YFL004W/VTC2 | 0.999 | caagcagcacgtgggtttttt | 1.28 | 0.77 | 1.39 | 1.60 | 2.03 | 0.23 | 1.71 | 1.58 |
| YHR136C/SPL2 | 1 | agcggagcacgtgggaaaaga | 2.45 | 3.72 | 4.65 | 4.61 | 1.52 | 3.01 | 5.25 | 3.09 |
| YHR215W/PHO12 | 1 | gcgttcacacgtgggtttaaa | 3.89 | 3.08 | 4.20 | 4.16 | 4.71 | 3.25 | 5.23 | 3.73 |
| YJL012C/VTC4 | 1 | tcatccgcacgtggctgcaca | 2.22 | 2.10 | 2.89 | 3.30 | 2.80 | 1.90 | 3.07 | 3.09 |
| YJL117W/PHO86 | 1 | gcgcccgcacgtgctctttat | 1.40 | 0.89 | 1.48 | 1.36 | 2.08 | 0.95 | 1.78 | 1.35 |
| YML123C/PHO84 | 1 | acacgtccacgtggaactatt | 3.30 | 5.09 | 5.34 | 5.49 | 3.72 | 3.53 | 5.37 | 2.78 |
| YPL018W/CTF19 | 1 | gagggcccacgtggcttaata | -0.12 | 1.86 | 1.92 | 1.86 | 1.77 | 1.63 | 2.10 | 0.51 |
| YPL019C/VTC3 | 1 | gagggcccacgtggcttaata | 3.02 | 3.47 | 3.94 | 4.30 | 4.04 | 2.25 | 4.09 | 2.70 |
| YOL084W/PHM7 | 1 | atgtgcgcaagtgcttagaaa | 1.35 | 2.33 | 2.31 | 0.12 | 1.06 | 1.29 | 0.99 | 1.16 |

*^*^ p* denotes the probability of being a target gene

*^†^* The set of microarray experiments are: *A*. Low-Pi vs High-Pi in WT (NBW7) exp1; *B.* Low-Pi vs High-Pi in WT (NBW7) exp2; *C.* Low-Pi vs High-Pi in WT (DBY7286); *D.* PHO4c vs WT; *E.* pho80 vs WT; *F.* pho85 vs WT; *G*. PHO81c vs WT exp1; and *H.* PHO81c vs WT exp2.
