# Supplementary material for: Identification of Direct Target Genes Using Joint Sequence and Expression Likelihood with Application to DAF-16
Source: PLoS One. 2008 Mar 19;3(3):e1821. doi: 10.1371/journal.pone.0001821 (PMC2266795; doi:10.1371/journal.pone.0001821)
Supplement: Table S2 — (0.10 MB DOC) [file pone.0001821.s005.doc]

Table S2. Class 1 ageing genes identified by TRANSMODIS

| Gene ORF | Gene Name | *P^*^* | Extended motif | Deviation contrast in log expression level comparing daf-2(RNAi) experiments to mixed timecourse data | Deviation contrast in log expression level comparing daf-16(RNAi):daf-2(RNAi) experiments to mixed timecourse data |
| --- | --- | --- | --- | --- | --- |
| T22G5.7 | spp-12 | 1 | actatcctgtttacttccaga | 1.63 | -1.66 |
| T20G5.7 | dod-6 | 1 | tgaaaaatatttacttaacat | 2.13 | -1.36 |
| C24B9.9 | dod-3 | 1 | gtgataatgtttaccccgcgg | 1.16 | -0.64 |
| F28D1.5 | thn-2 | 1 | aagatttttttttccaaaaaa | 1.54 | -0.54 |
| F48D6.4 | f48d6.4 | 1 | gttagtttattaacttagttt | 1.12 | -0.85 |
| F28D1.3 | thn-1 | 1 | gtgggtttgtttacagtcctt | 1.42 | -0.61 |
| Y40B10A.6 | y40b10a.6 | 1 | taattattatttactgagtaa | 2.16 | -1.95 |
| ZK384.1 | zk384.1 | 1 | tcaacaatgtttgcaactccg | 1.06 | -1.75 |
| T25C12.2 | spp-9 | 1 | aaaaaagtatttacccaaaag | 0.45 | -2.14 |
| PDB1.1 | pdb1.1 | 1 | ctttcattatttactctattc | 1.30 | -0.62 |
| ZK355.3 | zk355.3 | 1 | cacaaaaaatttacttcttgt | 1.84 | -1.06 |
| K12G11.3 | sodh-1 | 1 | cccaaaatgttttctgaacaa | -0.02 | -1.39 |
| C02A12.4 | lys-7 | 1 | tttatactgtttacttcagtg | 1.72 | -1.49 |
| R09B5.6 | hacd-1 | 1 | ccttttttgttaaccactttt | 1.19 | -0.80 |
| C55B7.4 | acdh-1 | 1 | ctgaaaatgtttatttcttga | 0.05 | -1.23 |
| K11G9.6 | mtl-1 | 1 | tgctggctgtttaccacttca | 1.61 | -2.82 |
| C54F6.14 | ftn-1 | 1 | gggttcttgtttacagaaaca | 1.91 | -0.96 |
| ZK384.2 | zk384.2 | 1 | ggtatgatattttctgaaatt | 0.90 | -0.69 |
| K07C6.4 | cyp-35b1 | 1 | acaaatttatttactaaaatc | 1.32 | -0.63 |
| B0213.15 | cyp-34a9 | 1 | tttataatttttacatttatt | 1.24 | -0.44 |
| C54D10.1 | cdr-2 | 1 | ttaaaactatttaaattcaaa | 1.21 | -0.53 |
| F11A5.12 | stdh-2 | 1 | cagatattattttcttcattc | 1.28 | -0.35 |
| W06D12.3 | fat-5 | 1 | ttttgtttatttacttaatta | 1.32 | -0.45 |
| T02B5.1 | t02b5.1 | 1 | tcatttttatttacatgtact | 1.49 | -0.12 |
| ZK384.3 | zk384.3 | 1 | gaaattatattttctattcca | 1.16 | -0.15 |
| C08E8.4 | c08e8.4 | 1 | gtgaccttgtttactgcctcc | 1.34 | -0.27 |
| C06B3.4 | stdh-1 | 1 | caaaatatatttacagacagt | 1.27 | -0.35 |
| B0286.3 | b0286.3 | 1 | atcattatattttcaaatttt | 1.37 | -0.19 |
| E01A2.8 | e01a2.8 | 1 | ggaaatatgtttactgtaaaa | 0.91 | -0.79 |
| C50F7.2 | clx-1 | 1 | cctcctttatttacattgacc | 1.30 | -0.16 |
| M02D8.4 | m02d8.4 | 1 | cgttgtgtgtttactttattg | 1.21 | -0.37 |
| C17G10.5 | lys-8 | 1 | atgataatgttttccgaaatt | 1.07 | -0.34 |
| F49A5.6 | thn-4 | 1 | atgttgatattttctttcttg | 1.20 | -0.24 |
| M01H9.3 | m01h9.3 | 0.998 | gtttgtctgtttccttcaaag | 1.39 | -0.06 |
| C52D10.1 | c52d10.1 | 0.992 | ataattttatttattgttttt | 1.22 | -0.26 |
| VZK822L.1 | fat-6 | 0.975 | tttatattattttctagaagc | 1.20 | -0.23 |
| C06B3.5 | c06b3.5 | 0.97 | gcgagaatatttactttttta | 1.10 | -0.22 |
| C05E4.9 | gei-7 | 0.898 | atgtaattgtttactcaactt | 1.14 | -0.36 |
| C30G12.2 | c30g12.2 | 0.776 | gaaaattcattaactgaaaca | 0.91 | -0.31 |

*^*^ p* denotes the probability of being a target gene
