# Supplementary material for: Identification of Direct Target Genes Using Joint Sequence and Expression Likelihood with Application to DAF-16
Source: PLoS One. 2008 Mar 19;3(3):e1821. doi: 10.1371/journal.pone.0001821 (PMC2266795; doi:10.1371/journal.pone.0001821)
Supplement: Table S3 — (0.32 MB DOC) [file pone.0001821.s006.doc]

Table S3. Class 2 ageing genes identified by TRANSMODIS

| Gene ORF | Gene Name | *P^*^* | Extended motif | Deviation contrast in log expression level comparing daf-2(RNAi) experiments to mixed timecourse data | Deviation contrast in log expression level comparing daf-16(RNAi):daf-2(RNAi) experiments to mixed timecourse data |
| --- | --- | --- | --- | --- | --- |
| C32H11.9 | c32h11.9 | 1 | Cttgtgatattcacaaagttt | -1.35 | 0.47 |
| K04E7.2 | opt-2 | 1 | ctcgttatgtttactgtgtgt | -0.63 | 0.82 |
| ZC513.11 | str-138 | 1 | ataaaaatatttcctaattta | -0.42 | 0.30 |
| ZC404.5 | srh-28 | 1 | cttctggtatttacaacatta | -0.71 | -0.35 |
| ZK6.7 | zk6.7 | 1 | gtctgtatgtttacttttggt | -0.40 | -0.20 |
| Y38H6C.5 | y38h6c.5 | 1 | ttcattttatatactaaattc | -0.71 | 0.49 |
| T02B11.5 | srj-38 | 1 | gtttttttgttttctgaaaat | -0.96 | -0.65 |
| C32H11.4 | c32h11.4 | 1 | agtcacatatttacaaagttc | -0.97 | 0.76 |
| ZK6.11 | zk6.11 | 1 | tttgcgacatttacagtttta | -1.56 | 0.82 |
| W05B2.6 | col-92 | 1 | tattgtttctttactatgttt | -0.42 | -0.62 |
| T25C12.3 | t25c12.3 | 1 | ttgggaatgtttacttgttgc | -0.59 | 0.72 |
| K09D9.2 | cyp-35a3 | 1 | tatgatatatttacagccccc | -0.95 | 0.33 |
| T10B5.4 | t10b5.4 | 1 | ttccaagtattgacattttcc | -0.68 | -0.45 |
| B0554.6 | dod-20 | 1 | tccaacttatgtacattaacg | -0.55 | 0.91 |
| C46E10.2 | c46e10.2 | 1 | gtctgcctatttacaagccag | -1.37 | -0.45 |
| C32H11.10 | dod-21 | 1 | gtatgcttttttactgtcaat | -1.99 | 0.18 |
| Y38H6C.20 | y38h6c.20 | 1 | ctttctttgttttctattttt | -1.33 | -0.62 |
| F55G11.7 | f55g11.7 | 1 | aatgacgaatttacaaatttt | -0.94 | 0.52 |
| C32H11.12 | dod-24 | 1 | atttagatattaactaaagat | -1.39 | 0.45 |
| K08D9.6 | k08d9.6 | 1 | ttcaaattattttcaagttac | -1.95 | -1.66 |
| F54B11.4 | f54b11.4 | 1 | tataaaatgtttagttaaaga | 0.06 | 1.00 |
| F28B4.3 | f28b4.3 | 1 | tttacgatatttagttttttt | -0.53 | 0.62 |
| W05B2.1 | col-94 | 1 | gcggaagtgtttacgatcggt | -0.33 | -0.72 |
| F35E12.5 | f35e12.5 | 1 | ctttatatttttattattggt | -0.64 | 0.69 |
| B0207.10 | b0207.10 | 1 | ttgaatttatttataattttt | -0.76 | -0.37 |
| F55G11.5 | dod-22 | 1 | cttaaaatgtttacaggtgga | -1.83 | 0.68 |
| C31A11.5 | c31a11.5 | 1 | tacgatatattttcaattatt | -0.61 | 0.38 |
| F15E11.12 | f15e11.12 | 1 | ctaaaaatatttacttgcctg | -0.51 | -0.64 |
| T24B8.5 | t24b8.5 | 1 | tttaaattgttttcatacttt | -0.67 | 0.20 |
| W05B2.5 | col-93 | 1 | ttatgcatgtttaaacatttc | -0.51 | -0.50 |
| F15E11.1 | f15e11.1 | 1 | tttaaaatggttaccgtatca | -1.00 | -0.31 |
| F56A4.2 | f56a4.2 | 1 | gataatatttttacataaata | -1.39 | 0.32 |
| F11G11.11 | col-20 | 1 | attgaattgtatacttttttt | -0.59 | -1.05 |
| H28G03.3 | h28g03.3 | 1 | aattttttatttactaatctt | -1.66 | -0.95 |
| F57F4.4 | f57f4.4 | 1 | aataaaatttttactttactg | -0.82 | 0.45 |
| T11F9.2 | tag-140 | 1 | cttcatatgtttaaaattttt | -0.96 | -0.76 |
| K10D11.1 | dod-17 | 1 | gcaaaattatttacacgtgtt | -1.20 | 0.50 |
| F46C8.6 | dpy-7 | 1 | tctgaaatgtgtacagttgca | -0.47 | -0.14 |
| F28H7.3 | f28h7.3 | 1 | tttcccatatttacatctcga | -0.87 | 0.28 |
| ZK1037.4 | nhr-246 | 1 | tcaataatgtttacaaaaatc | -1.25 | -0.93 |
| F22D6.10 | col-60 | 1 | tatcaagtttttacacaatca | -0.65 | -0.22 |
| F49E12.2 | dod-23 | 1 | caattcaaatttacagaaaat | -0.90 | 0.89 |
| T28H11.2 | srm-1 | 1 | tctgaaatatttaaaggtatt | -1.14 | -1.20 |
| F55G11.8 | f55g11.8 | 1 | ttctacgtattttcactcttt | -0.99 | 0.28 |
| T03D3.1 | ugt-53 | 1 | tactatgtgtttacacaaaaa | -0.55 | 0.81 |
| F44C4.3 | cpr-4 | 1 | tattctttttttacaacttca | -0.29 | 0.72 |
| T05E12.3 | t05e12.3 | 1 | cattttctttttacaaaaaat | -0.23 | 1.00 |
| F56G4.3 | f56g4.3 | 1 | aaaaaaatattaaccgttttt | -0.45 | 0.61 |
| F32A5.3 | f32a5.3 | 1 | ctttcatgatttacaggtttt | -0.51 | 0.76 |
| F49F1.1 | f49f1.1 | 1 | gttactttatttaaaaaattt | -0.29 | 0.66 |
| T20F10.4 | t20f10.4 | 1 | atcttggtatttacaattatt | -0.73 | 0.13 |
| M18.1 | col-129 | 1 | ctttgaatatttacaatttga | -0.26 | -0.41 |
| C32H11.13 | c32h11.13 | 1 | atttagatattaactaaagat | -0.23 | 0.66 |
| C15A11.5 | col-7 | 1 | ttatcaatatttattaattgc | -0.41 | -0.96 |
| C53B4.5 | col-119 | 1 | ttttttatatttgcttatcaa | -0.33 | -0.69 |
| F53A9.8 | f53a9.8 | 1 | tataaaatattaactgaagat | -0.18 | 0.90 |
| F11H8.3 | col-8 | 1 | ttagttttatttatttgttga | -0.15 | -0.37 |
| C34F6.2 | col-178 | 1 | ttttcagtaattacggtagaa | -0.14 | -0.26 |
| R04E5.10 | ifd-1 | 1 | atataaatattttctattaaa | -0.01 | 1.11 |
| C01H6.1 | col-61 | 1 | atctaaatatttacaaacaat | -0.29 | 0.59 |
| F55D12.6 | f55d12.6 | 1 | ttcaatttatttatgctttct | -0.52 | 0.11 |
| W02D3.7 | lbp-5 | 1 | tgtgtcctatttactatatat | -0.24 | 0.80 |
| F55G11.2 | f55g11.2 | 1 | tttttcgtgttttcaatttct | -0.30 | 0.78 |
| C31G12.4 | c31g12.4 | 1 | attaatttatttaacgtacta | -0.84 | -1.09 |
| C07B5.5 | nuc-1 | 1 | tttgctatgtttactaaaatg | -0.30 | 0.57 |
| D2023.7 | col-158 | 1 | tttaaactgttcacagatatt | -0.15 | -0.29 |
| ZK757.1 | zk757.1 | 1 | tttaccatatttacctctttt | -0.09 | 0.53 |
| R13H4.3 | r13h4.3 | 1 | ctagcattttttacaaagtta | -0.09 | 0.88 |
| F07C3.1 | ptd-2 | 1 | gatttcctgtttaaaattgtt | -0.54 | -0.53 |
| F29C12.1 | pqn-32 | 1 | gttctagtatctacaaaatta | 0.15 | 1.01 |
| F15H10.1 | col-12 | 1 | ttttcagtatttgctattgac | -0.15 | -0.42 |
| F10G8.3 | npp-17 | 1 | gttataatatttataatcaaa | -0.53 | -0.20 |
| F08G5.6 | f08g5.6 | 1 | atataaatatttaccatgtca | -0.15 | 1.17 |
| F40F4.6 | f40f4.6 | 1 | attttcttatttaacgacttt | -0.25 | 0.69 |
| C25D7.12 | c25d7.12 | 1 | cttgttttatttataatcggt | -0.28 | 0.19 |
| C17F3.3 | c17f3.3 | 1 | tttaaaaaatttacacaccaa | -0.17 | 0.65 |
| T10B9.2 | cyp-13a5 | 1 | accgacatatttaccaaggcc | -0.63 | -0.93 |
| F23B2.12 | pcp-2 | 1 | tgataactgtttagagatgtt | -0.01 | 0.78 |
| F35E12.9 | f35e12.9 | 1 | tgtgtaatattgacaaaaatt | -0.11 | 0.83 |
| C05E11.5 | amt-4 | 1 | ctttaagaatttacacctcac | -0.09 | 0.80 |
| F57B1.4 | col-160 | 1 | acggaactatttactgaaaac | -0.11 | -0.67 |
| K06H6.5 | k06h6.5 | 1 | ttctgtatatttaagattttt | -0.29 | -0.20 |
| F54F11.2 | f54f11.2 | 1 | ctaaaaatatttgccaaatac | -0.03 | 0.80 |
| Y62H9A.4 | y62h9a.4 | 1 | ttctgattgtttaaactttta | -0.14 | 0.60 |
| T11F9.9 | col-157 | 1 | ttggatatgtttcctataaaa | -0.08 | -0.39 |
| C08F11.8 | ugt-22 | 1 | gtgaaaatttttactgtttct | -0.04 | 0.59 |
| F11D11.8 | f11d11.8 | 1 | tttagaatgtttaaggaaaat | -0.29 | 0.09 |
| C54G4.2 | c54g4.2 | 1 | cttctcatatttataaaatga | -1.02 | -1.52 |
| W01A11.4 | lec-10 | 1 | ctttttgtatttccaaaatga | -0.24 | 0.38 |
| T25B9.7 | ugt-54 | 1 | ataaaattatttacagaaata | -0.46 | -0.34 |
| C14A6.1 | clec-48 | 1 | atttaattttttacacgatta | -0.03 | 0.35 |
| F55C7.2 | f55c7.2 | 1 | attttcatattttcagacgaa | 0.29 | 1.24 |
| Y106G6D.3 | y106g6d.3 | 1 | aactacctgtttactgtagtc | -0.11 | 0.75 |
| C43D7.5 | sdz-6 | 1 | gaaagcctgtttacggatgga | 0.32 | 1.14 |
| ZC416.6 | zc416.6 | 1 | agaaaagtatatacaaatcca | -0.14 | 0.87 |
| F59B2.13 | f59b2.13 | 1 | ctctttttatttacatttaaa | -0.31 | 0.04 |
| F10D11.6 | f10d11.6 | 1 | atcatcatatttccattgtcc | -0.33 | 0.17 |
| T09F5.7 | t09f5.7 | 1 | atccaattctttacatttggt | -0.10 | 0.05 |
| DY3.5 | pqn-26 | 1 | tctaaaatgtttaaaatttgt | 0.10 | 0.99 |
| C55B6.5 | c55b6.5 | 1 | ataagcatatttattgataga | 0.20 | 1.33 |
| W05E10.4 | tre-3 | 1 | tatgaaatatttattgatatc | -0.03 | 0.60 |
| C15A11.6 | col-62 | 0.999 | attaaagtatttaaaaaattt | -0.29 | -0.70 |
| B0379.2 | b0379.2 | 0.999 | ttataaatatttacgcaatat | -0.26 | 0.45 |
| Y41E3.2 | dpy-4 | 0.999 | ataaaaaaatttactgtttct | -0.40 | -1.05 |
| F56H9.1 | srx-113 | 0.999 | attccaatatttatttctgtt | 0.25 | 1.13 |
| F16C3.1 | f16c3.1 | 0.999 | ttttcggtgtttacacgtctt | -0.50 | 0.04 |
| F22A3.6 | f22a3.6 | 0.999 | gtttgattgtttactgttttg | -0.23 | 0.04 |
| F38B6.5 | col-172 | 0.999 | cctcacttatttacatttctt | 0.15 | 0.97 |
| C46H11.6 | c46h11.6 | 0.999 | ttttatatgtttatactttta | 0.03 | 0.77 |
| F56F4.7 | f56f4.7 | 0.999 | ataaaaacgtttacaaacctt | 0.04 | 1.04 |
| B0495.4 | nhx-2 | 0.999 | attaatatatatacttttttc | 0.01 | 0.80 |
| F33D11.6 | f33d11.6 | 0.998 | ttctagttatttacactttgg | -0.41 | -0.16 |
| C52D10.9 | skr-8 | 0.998 | aaaaaactgtttaaaatttat | -0.15 | 0.52 |
| F26B1.4 | col-58 | 0.997 | tttcagatatctactttttga | -0.37 | -0.65 |
| C24H10.3 | c24h10.3 | 0.996 | attagaatatttattgaactc | -0.29 | 0.18 |
| F52C9.5 | f52c9.5 | 0.996 | ttattcgtttttacttttgtg | -0.35 | -0.21 |
| K12G11.4 | sodh-2 | 0.996 | cttgaaatgtttagaattctc | -0.31 | -0.34 |
| D1007.2 | col-52 | 0.996 | ttttaagtgtttcaagttttt | -0.25 | 0.30 |
| C31H2.2 | dpy-8 | 0.994 | ttctgtatgtttattattttt | 0.01 | 0.25 |
| ZK1290.6 | zk1290.6 | 0.993 | tttaatctattttctcaagga | -0.03 | 0.67 |
| R09A8.4 | col-182 | 0.989 | tcttttttgtttaaattttta | -0.03 | 0.39 |
| C15A11.1 | col-35 | 0.988 | atttacatatttaatcttttc | -0.27 | -0.71 |
| F56G4.2 | pes-2 | 0.987 | atcattgtatttaccgtatcg | -0.31 | 0.44 |
| C24G7.1 | c24g7.1 | 0.98 | acttacttacttacagtagtt | 0.05 | 0.85 |
| K12H4.7 | k12h4.7 | 0.97 | gtttaattgtttactggaact | -0.15 | 0.25 |
| T22G5.2 | lbp-7 | 0.97 | ctgcaattttttacaaaaaat | -0.07 | 0.62 |
| E03A3.8 | e03a3.8 | 0.968 | tcgtaaatatttacttatttt | -0.26 | 0.21 |
| Y39D8A.1 | y39d8a.1 | 0.965 | gatattttatttacagtaccc | -0.01 | 0.81 |
| F56D5.1 | col-121 | 0.957 | aataaaatttttactatttta | 0.11 | 0.86 |
| C08B11.4 | nrf-6 | 0.946 | aattatataattacagtactc | 0.19 | 0.96 |
| C09G5.8 | c09g5.8 | 0.944 | aattttatatttccttttttc | -0.13 | 0.43 |
| B0393.7 | b0393.7 | 0.941 | attcaattaattacaattcat | -0.12 | 0.50 |
| C05A9.1 | pgp-5 | 0.94 | tttaccgtgtttacttaataa | 0.46 | 1.23 |
| C46H11.1 | c46h11.1 | 0.938 | ttagttatgtatacaaatact | 0.06 | 0.73 |
| C55B7.4 | acdh-1 | 0.914 | ctgaaaatgtttatttcttga | 0.05 | -1.23 |
| R09B5.2 | cnc-1 | 0.912 | tatgatttgttttcatttaat | 0.16 | 0.82 |
| R10E11.7 | srxa-10 | 0.91 | caagagctatttacacttctg | -0.39 | 0.12 |
| W01B11.2 | sulp-6 | 0.832 | atttacatattttcaaataaa | 0.12 | 0.75 |
| C10A4.7 | c10a4.7 | 0.801 | actcaactatttagttttgac | -0.02 | 0.69 |
| W06D4.2 | w06d4.2 | 0.763 | ttccatgtgttaacaataaat | 0.17 | 0.72 |
| C25A1.15 | c25a1.15 | 0.754 | ctgaaattatttaaaatttaa | -0.03 | 0.40 |
| F55F8.7 | f55f8.7 | 0.736 | tttcaaatatttccaaaaatt | 0.20 | 1.00 |
| F55D12.4 | unc-55 | 0.724 | ctgatcataattacaatattt | -0.12 | 0.50 |
| B0379.6 | b0379.6 | 0.677 | tattttttgtttaccacacac | -0.22 | 0.41 |
| F46B3.7 | f46b3.7 | 0.661 | aatgatcaatttacagatgca | 0.03 | 0.60 |
| C46H11.7 | c46h11.7 | 0.647 | ctaaaactgtatacatttttt | -0.31 | 0.21 |
| T28F2.4 | t28f2.4 | 0.617 | ttttttttattaacataagta | 0.05 | 0.93 |
| C42C1.7 | c42c1.7 | 0.534 | ttttaaatgtttaaaaatttt | 0.07 | 0.72 |
| M04C9.4 | m04c9.4 | 0.528 | gtttaaatattttcaattcga | 0.17 | 0.96 |
| T16G12.1 | t16g12.1 | 0.507 | ataataatatttaattaatta | 0.00 | 0.56 |

*^*^ p* denotes the probability of being a target gene
