# Supplementary material for: Identification of Direct Target Genes Using Joint Sequence and Expression Likelihood with Application to DAF-16
Source: PLoS One. 2008 Mar 19;3(3):e1821. doi: 10.1371/journal.pone.0001821 (PMC2266795; doi:10.1371/journal.pone.0001821)
Supplement: Table S4 — (0.02 MB DOC) [file pone.0001821.s007.doc]

Table S4. Overall agreement between the two strategies of updating $\sigma_{1}$ (calculated in terms of Cohen’s kappa)

|  | *μ_1_* = 1 | 2 | 5 |
| --- | --- | --- | --- |
| *σ_1_^2^* = 0.5 | 0.9221±0.0940 | 0.9970±0.0046 | 1.000±0.0000 |
| 1 | 0.9118±0.1010 | 0.9917±0.0095 | 0.9999±0.0008 |
| 2 | 0.9559±0.0636 | 0.9876±0.0137 | 0.9998±0.0010 |
