# Supplementary material for: Identification of Direct Target Genes Using Joint Sequence and Expression Likelihood with Application to DAF-16
Source: PLoS One. 2008 Mar 19;3(3):e1821. doi: 10.1371/journal.pone.0001821 (PMC2266795; doi:10.1371/journal.pone.0001821)
Supplement: Text S1 — Supplementary text (0.24 MB DOC) [file pone.0001821.s008.doc]

**Details of TRANSMODIS**

**The parametric model and the EM algorithm**

The parametric model of TRANSMODIS contains an expression and a sequence component. Target genes are assumed to differ from non-targets in both expression levels and patterns of extended motifs (i.e., the core motif along with immediate flanking regions). The expression of targets and non-targets is modeled by a two-component Gaussian mixture distribution, and the nucleotide frequencies at each position of the extended binding motif are assumed to be multinomial which is represented by a position specific weight matrix (PSWM). Presumably, non-targets do not have binding sites and their sequences are drawn from a background PSWM.

The model is fitted to the sequence and expression data by maximizing the observed log-likelihood function,$LL_{o}=\log P(S,E;\theta)$, where *S* and E denote the sequence and expression data respectively, and *θ* denotes the collection of all model parameters. After expansion into a sum,

$LL_{0}=\sum_{i=1}^{N} \log\left[\left(\prod_{k=1}^{W} f_{a_{k}(i),k}\right)\left(\prod_{j=1}^{M} \frac{1}{\sqrt{2\pi}\sigma_{1}}e^{-\frac{(E_{ij}-\mu_{1})^{2}}{2\sigma_{1}^{2}}}\right)\lambda+\left(\prod_{k=1}^{W} f_{_{a_{k}(i),k}}^{0}\right)\left(\prod_{j=1}^{M} \frac{1}{\sqrt{2\pi}\sigma_{0}}e^{-\frac{(E_{ij}-\mu_{0})^{2}}{2\sigma_{0}^{2}}}\right)(1-\lambda)\right],$ where λ is the proportion of true targets among all genes; $\mu_{1}$ and $\sigma_{1}$ denote the mean and the standard deviation of the target expression distribution; *N* and *M* denote the total number of genes and experiments respectively; $f_{jk}$ and $f_{jk}^{0}$ denote the *(j,k)^th^* entries of target and non-target PSWMs, or the probabilities of observing the *j^th^* alphabet at position *k* in the target and non-target sequence; *A* is the size of the sequence alphabet (e.g., *A*=4 for DNA sequences); *W* is the length of extended motifs; and $a_{k}(i)$ returns the alphabet of the *i*^th^ sequence at position *k*. When *M=*1, i.e. there is a single expression profile, TRANSMODIS reduces to MODEM and the observed log-likelihood function shown above becomes that of the mixture model in MODEM.

Direct maximization of *LL_o_* is a formidable task. Therefore an expectation-maximization (EM) algorithm was derived to compute maximum likelihood estimates (MLEs). The EM algorithm is a generic iterative algorithm for parameter estimation by maximum likelihood principle. Each iteration of an EM algorithm involves two steps: an E-step where the latent variables are imputed by their expectations and an M-step where the complete log-likelihood function is maximized. In our model, the latent variables are the membership variables. Define $Z=(Z_{1},\ldots,Z_{N})$, where

$$Z_{i}=\left\{\begin{matrix} 1,\text{ if gene }i\text{ is a true target} \\ 0,\text{ otherwise. } \end{matrix}\right.$$

The complete log-likelihood $LL_{c}$ is given by $\log P(S,E,Z;\theta)=\sum_{i=1}^{N} \log P(S_{i},E_{i\cdot},Z_{i};\theta)$, where $E_{i\cdot}$ denotes the expression profile of gene *i*, i.e., the *i*^th^ row of expression matrix *E*. Because *Z_i_* is a binary random variable, we have

$$\log P(S_{i},E_{i\cdot},Z_{i};\theta)=\log[P(S_{i},E_{i\cdot}|Z_{i};\theta)P(Z_{i};\theta)]$$

$$=Z_{i}\log[P(S_{i},E_{i\cdot}|Z_{i}=1;\theta)P(Z_{i}=1;\theta)]+(1-Z_{i})\log[P(S_{i},E_{i\cdot}|Z_{i}=0;\theta)P(Z_{i}=0;\theta)]$$

The M-step involves the maximization of function $Q(\theta,\hat{\theta})$, which is, by definition, $E_{Z|S,E;\hat{\theta}}(LL_{c})$:

$$Q(\theta,\hat{\theta})=E_{Z|S,E;\hat{\theta}}(LL_{c})$$

$$=\sum_{i=1}^{N} \{(E_{Z_{i}|S_{i},E_{i\cdot};\hat{\theta}}(Z_{i}))\log[P(S_{i},E_{i\cdot}|Z_{i}=1;\theta)P(Z_{i}=1;\theta)]+$$

$$(1-E_{Z_{i}|S_{i},E_{i\cdot};\hat{\theta}}(Z_{i}))\log[P(S_{i},E_{i\cdot}|Z_{i}=0;\theta)P(Z_{i}=0;\theta)]\}$$

where $\hat{r}_{i}=E_{Z_{i}|S_{i},E_{i\cdot};\hat{\theta}}(Z_{i})$. In words, $\hat{r}_{i}$ is the probability of gene *i* being a target given the observed data, *S_i_* and $E_{i\cdot}$, under the current estimate $\hat{\theta}$.

The function can be re-written as a sum of three parts:

$$Q(\theta,\hat{\theta})=$$

The three addends are the log-likelihoods from the mixing proportion, the sequence data and the expression data respectively. After taking the partial derivatives of with respect to each unknown model parameter and setting them to zero, we obtain the following updating formulas:

(S1)

(S2)

(S3)

$1\leq j\leq A,$ $1\leq k\leq W$ (S4)

$1\leq j\leq A,$ $1\leq k\leq W,$ (S5)

where $I(\cdot)$ is the indicator function.

**Computation of *r_i_*’s**

By definition, $\hat{r}_{i}=E_{Z_{i}|S_{i},E_{i\cdot};\hat{\theta}}(Z_{i})=\Pr(Z_{i}=1|S,E;\hat{\theta})$. Using Bayes’ theorem, we have

$\Pr(Z_{i}=1|S,E;\hat{\theta})=\frac{\Pr(S,E|Z_{i}=1;\hat{\theta})\Pr(Z_{i}=1;\hat{\theta})}{\Pr(S,E|Z_{i}=1;\hat{\theta})\Pr(Z_{i}=1;\hat{\theta})+\Pr(S,E|Z_{i}=0;\hat{\theta})\Pr(Z_{i}=0;\hat{\theta})}$ (S6)

Even though the formula in (S6) is correct analytically, $P(S_{i},E_{i\cdot}|Z_{i}=1;\hat{\theta})$ and $P(S_{i},E_{i\cdot}|Z_{i}=0;\hat{\theta})$ are typically very small quantities such that a direct computation would cause numerical underflow. Therefore, TRANSMODIS computes the probabilities $\hat{r}_{i}$s, by the following equivalent equation to avoid underflow:

$\hat{r}_{i}=\frac{\hat{\lambda}}{\hat{\lambda}+(1-\hat{\lambda})\exp[\log P(S_{i},E_{i\cdot}|Z_{i}=0;\hat{\theta})-\log P(S_{i},E_{i\cdot}|Z_{i}=1;\hat{\theta})]}$ (S7)

**Initialization of parameters**

Each experiment profile is standardized to have mean zero and standard deviation one. This is a necessary data-adjustment step to correct array bias that arises from variation in the technology rather than variation in the biology. The parameters for the baseline Gaussian component, $\mu_{0}$ and $\sigma_{0}$, are set to zero and one respectively throughout the iterations.

Initially, nucleotide frequencies at each position (i.e., entries in the PSWMs) for targets and non-targets are both set to the overall observed frequencies in all sequences. The PSWMs start to diverge during subsequent iterations once they are updated.

The default initial values for the other parameters in the model, $\lambda$, $\mu_{1}$ and $\sigma_{1}$, are chosen to be 0.2, 2 and 0.5 respectively.

**Convergence criterion**

Convergence is considered to be achieved if the difference between two consecutive iterations of each parameter is less than a prescribed threshold. In particular, the set of convergence criteria used was: $|\lambda^{(k)}-\lambda^{(k-1)}|<0.01$, $|\mu_{1}^{(k)}-\mu_{1}^{(k-1)}|<0.05$, $|\sigma_{1}^{(k)}-\sigma_{1}^{(k-1)}|<0.01$, $|f_{jk}^{(k)}-f_{jk}^{(k-1)}|<0.02$ and $|f_{jk}^{0(k)}-f_{jk}^{0(k-1)}|<0.02$ for all $1\leq j\leq A$ and $1\leq k\leq W$.

**Safe-guarding**

When fitting a two component Gaussian mixture model to expression data, one needs to be cautious not to misinterpret conditional likelihood ratios. Denote fitted target and non-target distributions by $N(\mu_{1},\sigma_{1}^{2})$ and $N(\mu_{0},\sigma_{0}^{2})$ respectively. Without loss of generality, suppose that $\mu_{1}>\mu_{0}$, then there are two scenarios where a direct interpretation of the conditional likelihood ratios can be misleading:

1. When $\sigma_{1}>\sigma_{0}$, for a very negative expression value *e*, the ratio of probabilities of observing *e* from the non-target distribution over the target distribution (i.e. $\Pr(e|e\sim N(\mu_{0},\sigma_{0}^{2}))/\Pr(e|e\sim N(\mu_{1},\sigma_{1}^{2}))$) can be less than one, implying that the gene with an expression level of *e* is more likely to be a target than a non-target. However intuition tells us that actually the opposite is true, that is, negative expressions are more likely to be observed from non-target genes than from target genes (Figure S1).
2. Similarly, when $\sigma_{1}<\sigma_{0}$, for a very positive expression *e*, the probability ratio $\Pr(e|e\sim N(\mu_{0},\sigma_{0}^{2}))/\Pr(e|e\sim N(\mu_{1},\sigma_{1}^{2}))$ can be much greater than one, leading to the wrong interpretation that the gene is a non-target (Figure S1).

The underlying cause in both cases is an unequal variance between the target and non-target distributions. The implication is that using a two-component Gaussian mixture model to describe real expression data is inadequate. Nonetheless the normal mixture model is an analytically simple yet powerful parametric model to summarize expression data, under which MLEs can be computed via an EM algorithm. One simple remedy for the problem is to constrain the two variances to be equal, i.e., $\sigma_{1}=\sigma_{0}$. However we opted for a second solution: whenever an expression $e<\mu_{0}$ is observed, we required the ratio $\Pr(e|e\sim N(\mu_{0},\sigma_{0}^{2}))/\Pr(e|e\sim N(\mu_{1},\sigma_{1}^{2}))$ to be bounded below by $\Pr(e|e\sim N(\mu_{0},\sigma_{0}^{2}))/\Pr(e|e\sim N(\mu_{1},\sigma_{0}^{2}))$ (i.e. the probability ratio after substituting $\sigma_{1}$ for $\sigma_{0}$ as if the target distribution has variance $\sigma_{0}^{2}$). And similarly when $e>\mu_{1}$, the conditional likelihood ratio $\Pr(e|e\sim N(\mu_{0},\sigma_{0}^{2}))/\Pr(e|e\sim N(\mu_{1},\sigma_{1}^{2}))$ is restrained from exceeding $\Pr(e|e\sim N(\mu_{0},\sigma_{0}^{2}))/\Pr(e|e\sim N(\mu_{1},\sigma_{0}^{2}))$. We call our approach safe-guarding because it guards against a conditional likelihood ratio falling into an undesirable range.

**Outlier detection and removal**

The EM algorithm is sensitive to outliers. For example, imagine a non-target gene having all expression values close to $\mu_{0}$ except for one value which is very large ($\gg\mu_{1}$) due to some experimental error. This single spurious measurement can cause a large deviation in the computed probability and thus make the non-target gene falsely identified as a target gene by the EM algorithm. Because such outliers are detrimental to the analysis, they are searched for by TRANSMODIS and removed once found.

Under our expression model, the distribution of expression measurements of any given gene, whether a target or not, is Gaussian. Therefore the largest expression value of each gene is examined by comparing it with the rest of the gene’s expression measurements to see if it is probable to obtain an extreme value as large as the observed maximum. More precisely, let $E_{iJ}=\max_{j=1\ldots M} E_{ij}$ be the maximal expression value observed for gene *i*. A Gaussian density function is then fitted to the remaining (*M*-1) values: $\hat{\mu}=\sum_{j\neq J} E_{ij}/(M-1)$ and (if $M\geq3$). Assuming that all of the *M* expression values were drawn from this fitted Gaussian distribution, , then the probability of observing a maximum order statistics as large as $E_{iJ}$ is given by

, (S8)

where function $\Phi$ is the cumulative distribution function (cdf) of a standard normal. The entry $E_{iJ}$ is removed (i.e., treated as if it were missing) if the probability in (S8) is less than $\alpha$, a user-specified threshold (by default, $\alpha=0.05$).

The outlier detection and removal scheme described above is consistent with our parametric expression model and can remove up to one outlier per gene.

**A robust updating formula for** $\sigma_{1}$

Under our expression model, all target genes have the same average expression level. However in reality, this expression model is too simple to hold up. More likely, target genes have different baseline levels of expression. In other words, some target genes might be consistently more over-expressed than others even though all target genes are over-expressed in all experiments. Therefore it is sensible to subtract the baseline expression level of each gene when estimating $\sigma_{1}$, the standard deviation of gene expressions of a target,

(S9)

where $\bar{E}_{i\cdot}=\sum_{j=1}^{M} E_{ij}/M$ is the average expression of gene *i*. If the true underlying generative model is genuinely a two-component Gaussian mixture model, then replacing Equation (S3) by Equation (S9) has a negligible effect on the EM algorithm as both formulas give unbiased estimates of $\sigma_{1}$. However when the true underlying model deviates away from a two-component Gaussian mixture model, e.g., target genes do display different baseline levels of expression, then Equation (S9) produces a smaller estimate of $\sigma_{1}$ than Equation (S3). This downward biase has a beneficiary effect in controlling false positive rate because the probability of mistaking a non-target gene (assuming whose expression is below *μ_1_*) as a target steadily decreases as *σ_1_^2^* shrinks. Consequently, by having fewer non-target genes falsely classified as targets, the estimated value of *μ_1_* is less likely to shift unduly downward during subsequent iterations.

To verify that the substitution of Equation (S3) by Equation (S9) has minimal effect on target gene selection when the true generative model is indeed a two-component Gaussian mixture model, we carried out a simulation study, in which the targets’ expression distribution was one of the nine normal distributions (*μ_1_* = 1, 2 or 5, and *σ_1_^2^* = 0.5, 1 or 2, a total of nine combinations) while the non-targets’ expressions were all simulated from $N(0,1)$. For each target distribution, a total of 100 data sets were generated. Each simulated data set consists of 100 target genes and 900 non-target genes. To remove information from sequence data, all simulated genes had identical promoter sequences. Simulation results showed that by using either formula to update $\sigma_{1}$, the resultant target lists were always nearly identical (Table S4). We also compared the sensitivity and specificity of two target lists and found that using Equation (S3) resulted in a higher sensitivity while using Equation (S9) resulted in a higher specificity, but the differences were small and negligible (Figures S2 and S3). Because robustness was given a higher priority over sensitivity, TRANSMODIS updates its $\sigma_{1}$ estimate by using Equation (S9).

**Dealing with missing expression values**

It is common for microarray data to have missing entries. Many methods would require the missing entries to be imputed first; however imputation is optional with TRANSMODIS. In the presence of missing data, TRANSMODIS derives an EM algorithm that maximizes the likelihood on the available expression data entries only.
